# The optimal treatment of an infectious disease with two strains

**DOI:** 10.1007/s00285-016-1074-5

**Published:** 2016-11-11

**Authors:** Robert Rowthorn, Selma Walther

**Affiliations:** 1 Faculty of Economics, University of Cambridge, Cambridge, UK; 2 Department of Economics, University of Warwick, Coventry, CV4 7AL, UK

**Keywords:** Multiple strain infection, Economic epidemiology, Treatment, Optimal control, 91B55, 91B76, 93C15

## Abstract

This paper explores the optimal treatment of an infectious disease in a Susceptible-Infected-Susceptible model, where there are two strains of the disease and one strain is more infectious than the other. The strains are perfectly distinguishable, instantly diagnosed and equally costly in terms of social welfare. Treatment is equally costly and effective for both strains. Eradication is not possible, and there is no superinfection. In this model, we characterise two types of fixed points: coexistence equilibria, where both strains prevail, and boundary equilibria, where one strain is asymptotically eradicated and the other prevails at a positive level. We derive regimes of feasibility that determine which equilibria are feasible for which parameter combinations. Numerically, we show that optimal policy exhibits switch points over time, and that the paths to coexistence equilibria exhibit spirals, suggesting that coexistence equilibria are never the end points of optimal paths.

## Introduction

Several infectious diseases that pose a pressing problem in developing countries exist in multiple strains, including tuberculosis, HIV, Lyme disease, Human Papillomavirus, Helicobacter pylori and influenza (Balmer and Tanner 2011). Where resources are limited, a uniform treatment policy at the national level may be the most efficient way to tackle these diseases. This paper addresses the following question: what is the optimal treatment policy for an infectious disease that has two strains, which differ in their infectiousness? We take a standard model in epidemiology, the Susceptible-Infected-Susceptible (SIS) model, and extend it to include two strains of infection with different transmission probabilities. In the standard SIS model, individuals move between two states, susceptible and infected, based on exogenous probabilities. The probability of an individual catching a disease when he encounters an infected person depends on an infectivity or transmission parameter. This parameter is usually assumed to be homogeneous, which does not allow for policy differentiation if the disease exists in several strains.

The SIS model is often used to describe sexually transmitted diseases such as chlamydia. *Chlamydia trachomatis* is an organism that has three human strains: one strain causes *trachoma*, which is an infection of the eyes; a second strain causes sexually transmitted urethritis, often referred to simply as *chlamydia*, while a third strain causes the sexually transmitted *lymphogranuloma venereum* (*LGV*) (Byrne 2010). Chlamydia, caused by serovars D-K, is also the most common sexually transmitted disease in the United States and the United Kingdom, with over 1.2 million infections detected in the former and over 200 thousand in the latter.^1^ It can result in serious complications, such as pelvic inflammatory disease, which can lead to infertility if left untreated (Shaw et al. 2011). There are currently no vaccines for chlamydia, and treatment with antibiotics, usually with azithromycin or doxycycline, is necessary (Shaw et al. 2011). LGV is also treated with doxycycline (McLean et al. 2007), while trachoma is treated with azithromycin (Tabbara et al. 1996). Therefore, the optimal treatment mix for the various strains of chlamydia trachomatis is an important public health concern. It has been established in the literature that LGV strains are more infectious than trachoma strains, and that there may even be variation in virulence among trachoma strains (Pearce and Prain 1986; Kari et al. 2008). Reinfection with chlamydia is possible and superinfection does not appear to occur in practise (Heijne et al. 2011; Heiligenberg et al. 2014). Therefore, chlamydia trachomatis satisfies the assumptions of the model analysed in this paper, and as such, the results can provide guidance on the optimal treatment of the diseases that it causes.

We analyse the optimal treatment policy of a two-strain SIS disease, both analytically and numerically. We assume that the two strains differ in their infectivities, and neither superinfection nor eradication of the disease is possible. The policymaker can perfectly observe and target both strains with two separate treatment instruments. First, we provide theoretical results on the possible equilibria of this model. There are two sets of equilibria: non-boundary equilibria, where the two strains co-exist, and boundary equilibria, where one of the strains is endemic while the other is eliminated asymptotically. The feasibility of these equilibria depends on parameter conditions. Broadly speaking, when the infectivities of the two strains are very different, only the boundary equilibria can be achieved, and in particular those where the more infective strain prevails and the less infective strain is asymptotically eradicated. When the strains are quite similar, all equilibria are feasible. We demonstrate that in the coexistence equilibria, the more infective strain must receive more treatment than the less infective strain. These results lie in contrast to the equilibrium with no intervention, when the less infectious strain dies out.

We provide numerical examples where we simulate the model and look for optimal treatment policy along the path to the fixed points. We find that policy exhibits switch points along the path, implying that policy flexibility is beneficial, and that a static model would be too restrictive. We are unable to find a path towards a coexistence equilibrium that is optimal, in the sense of not being a spiral. We tentatively conclude that the coexistence equilibria can never be the end points of optimal paths, and that the boundary equilibria are always the optimal ones.

The ideas in this paper relate closely to the theory of competitive exclusion in ecology, which postulates that the coexistence of two or more species limited by one resource (in our case, two strains limited by one host population) is not possible (Armstrong and McGehee 1980). For example, Iggidr et al. (2006) demonstrate in the context of multiple-strain malaria that an equilibrium is either disease-free or has at most one surviving strain.

However, this is not always the case, provided some assumptions are relaxed, such as allowing for cycles in the persistence of species (Armstrong and McGehee 1980). In a model of renewable natural resources, coexistence of species is possible, with the stocks of the two resources higher in a mutualistic than a competitive equilibrium (Ströbele and Wacker 1991). In the case of an SIS model, coexistence of two strains is possible if one allows for superinfection and age structure (Li et al. 2010). Elbasha and Galvani (2005), in a model of two-strain Human Papillomavirus vaccines, show that when individuals are vaccinated for only one of the two strains, the equilibrium depends on whether the strains are synergistic (in which case vaccination also reduces the prevalence of the non-targeted strain) or antagonistic (where vaccination increases the prevalence of the other strain). Castillo-Chavez et al. (1999) demonstrate in a two-strain two-sex SIS model of a sexually transmitted disease that a unique coexistence equilibrium can occur under certain conditions. We show that in our two-strain SIS model, it is sometimes feasible for both strains to coexist, especially if they are not too different in their infectiousness. If one strain is much more infectious than the other, then it is only feasible to strive for the eradication of the less infectious strain, while allowing the more infectious strain to be endemic.

This paper builds on the economic epidemiology literature, which examines optimal intervention in tackling infectious diseases. Rowthorn et al. (2009) examine the optimal way to treat a disease with spatial dynamics, while Rowthorn (2006) considers the optimal treatment of a disease when policymakers have limited funds. There are important tradeoffs between treatment and vaccination as two instruments available to a policymaker (Rowthorn and Toxvaerd 2015). In general, a lack of intervention yields to inefficiently low uptake of vaccination (Chen and Toxvaerd 2014). While there is a rich literature that describes disease dynamics in the absence of intervention, the study of *optimal intervention* is still in its early stages, and this paper contributes to that literature.

The biology literature has shown the pervasiveness of diseases that exist in multiple strains, for which treatment and vaccination may have to vary. Lyme disease has at least 14 known strains.^2^ Seinost et al. (1999) present strong evidence that strains of Lyme disease have different infectivities. Other multiple-strain infections include Human Papillomavirus, which includes high-risk strains that are a leading cause of cervical cancer and low-risk strains that do not cause cancer, as well as tuberculosis, where some strains are particularly drug resistant (Castillo-Chavez and Feng 1997).^3^ Influenza is a multiple-strain disease: Truscott et al. (2012) argue that accurate modelling of influenza should not neglect the presence of several strains. HIV also exists in two forms: HIV-1 and HIV-2. Studies show that the less common HIV-2 strain is also less infectious than its counterpart for most of its infectious period.^4^ Accurate measurement of the transmission rate is the key to realistic simulations of HIV/AIDS prevalence rates for the USA and Africa (Oster 2005), which highlights both the importance of the transmission rate in describing an infection and the particular relevance of this paper to developing countries.

The paper proceeds as follows. Section 2 provides an overview of some theoretical concepts while Sect. 3 outlines the SIS model with two strains. Section 4 discusses the equilibria of the model and Sect. 5 provides examples of simulations. Section 6 concludes.

## Relevant concepts

It is useful to review some relevant concepts before the central analysis of the paper. The SIS model belongs to the category of transmission system models, which are built on differential equations that describe the evolution of disease prevalence over time as a function of parameters. It has become standard to assume random mixing in models of this type: agents are equally likely to encounter any other agent in the population.^5^ There are two possible states: individuals are either susceptible or infected. They can move between the two states an unlimited number of times. Agents are homogeneous and the population is closed. If a susceptible agent encounters an infected agent, the susceptible agent becomes infected with a certain probability, termed the transmission probability. Typically, these models assume a homogeneous transmission parameter for all agents. The standard SIS model is in continuous time. At every time increment, a proportion of infected individuals is treated with a certain success rate of treatment (which can be interpreted as a rate of recovery). There is also the possibility of spontaneous or natural recovery.

Our model differs from the standard SIS model in two key ways: the disease has two strains with distinct transmission rates and the policymaker has two policy instruments. We make three further assumptions about the nature of the disease: transmission is through human-to-human contact rather than vector-borne, superinfection is not possible and agents can recover fully from the disease. SIS models are often used to describe sexually transmitted diseases (Keeling and Eames 2005), and chlamydia is an appropriate example of an SIS disease with multiple strains (Byrne 2010).

Optimal policy determines what proportion of individuals to treat at each point in time subject to the cost of treatment, the value of susceptible and infected individuals to social welfare, as well as the transmission dynamics. Studies focus on deriving equilibria and looking for policy switches over time. Policy generally takes the form of a Most Rapid Approach Path (MRAP)—the policy that takes the system the fastest way to the desired equilibrium. Another point of interest is Skiba points or curves, which delineate points where the policymaker is indifferent between policies.^6^

An analysis of optimal treatment in the one-strain SIS model can be found in Rowthorn (2006) and Goldman and Lightwood (2002). We summarise their results here as a brief indication of what one might expect from a two-strain model. Two types of equilibria of the model exist: the policy instrument is either at a boundary, where no one or everyone is treated, or policy is interior, with partial treatment of the population. It is never optimal to treat partially; it can be shown that optimal policy will always take on one of the two boundary values. Whether it is optimal to treat everyone or no one depends on the parameters at hand.

## The SIS model with two strains

The model has the following characteristics. The two variants of the infection are High (*H*, high infectivity) and Low (*L*, low infectivity). The more infectious variant *H* has transmission rate $\beta _{H}$ while the less infectious variant *L* is characterised by transmission rate $\beta _{L}$.^7^ The two policy instruments are $f_{H},f_{L}\in [0,1]$, which measure the proportion of the infectees in each strain that are treated. The success rate of treatment is $\alpha \in (0,1]$. We assume that the policymaker has perfect information about the prevalence of each strain in the population and can therefore target treatment of each strain perfectly.^8^

Individuals can catch either strain at the outset. When infected, they transmit the strain that they themselves are infected with. There is a possibility of exogenous recovery at rate $\tau \in (0,1]$. If individuals recover, they are again susceptible to either infection strain. The proportion of the total population infected with the High strain is $I_{H}$. The proportion infected with the Low strain is $I_{L}$. To ease calculations, the total population is normalised to size 1: $I_{H}+I_{L}+S=1$, where *S* denotes the population of healthy individuals (susceptibles). All parameters are strictly positive. The policymaker places value *p* on healthy individuals, value 0 on infected individuals and discounts the future at rate $\delta$. She also faces a constant marginal cost *c* of treatment.

In order to choose the optimal policy mix of $f_{H}$ and $f_{L}$, the policymaker maximises the social welfare function

1 $$\begin{aligned} V(I_{H}^{0},I_{L}^{0})=\int _{0}^{\infty }e^{-\delta t}(p*(1-I_{H} (t)-I_{L}(t))-c*(f_{H}(t)I_{H}(t)+f_{L}(t)I_{L}(t)))dt, \end{aligned}$$

subject to the equations of motion for the two infection types:

2 $$\begin{aligned} \dot{I}_{H}= & {} I_{H}(t)\left[ \beta _{H}(1-I_{H}(t)-I_{L}(t))-\tau -\alpha f_{H}(t)\right] , \end{aligned}$$

3 $$\begin{aligned} \dot{I}_{L}= & {} I_{L}(t)\left[ \beta _{L}(1-I_{H}(t)-I_{L}(t))-\tau -\alpha f_{L}(t)\right] . \end{aligned}$$

Thus, the population of individuals infected with *H* increases at rate $\beta I_{H}S$, which is a direct consequence of the assumption of random mixing, and decreases at rate $I_{H}(\tau +\alpha f_{H})$. The population of agents infected with *L* changes in a symmetric way. We further assume that4Thus, we assume that the population begins with a positive level of prevalence of each strain. Assumption (4) states that the infection probability in an encounter between a healthy individual and a sick individual is higher than the probability of a treated sick individual recovering. This ensures that neither variant of the disease can be eliminated, even asymptotically, by treating all infected people (see Sect. 4.4 for a proof of this). Thus, $I_{H}(t),I_{L}(t)>0$ for all *t*.

In order to provide a benchmark for the optimal policy analysis, we discuss two simplifications of the model. First, we consider what happens when the two strains are independent, so that agents can be infected with both strains i.e. superinfection is possible, and infection with one strain has no impact on infection with the other strain. In this case, the disease dynamics are governed by the following equations:

$$\begin{aligned} \dot{I}_{H}&=\beta _{H}I_{H}(t)(1-I_{H}(t))-I_{H}(t)\tau -I_{H}(t)\alpha f_{H}(t),\\ \dot{I}_{L}&=\beta _{L}I_{L}(t)(1-I_{L}(t))-I_{L}(t)\tau -I_{L}(t)\alpha f_{L}(t). \end{aligned}$$

The equilibrium prevalence of the two strains is

$$\begin{aligned} I_{H}^{*}&=1-\frac{\alpha f_{H}+\tau }{\beta _{H}},\\ I_{L}^{*}&=1-\frac{\alpha f_{L}+\tau }{\beta _{L}}. \end{aligned}$$

Thus, prevalence depends only on the infectiousness of that strain and the extent to which it is treated. For the same level of treatment, the total number of infections (where a person infected with both strains counts as two infections) is higher than in the case when superinfection is not possible.

Second, we consider what happens to the infection when no intervention is possible; that is, $f_{H}=f_{L}=0$ at all points in time (but the infections are no longer independent). In this case, the two Eqs. (2) and (3) become

$$\begin{aligned} \dot{I}_{H}&=\beta _{H}I_{H}(t)(1-I_{H}(t)-I_{L}(t))-I_{H}(t)\tau ,\\ \dot{I}_{L}&=\beta _{L}I_{L}(t)(1-I_{H}(t)-I_{L}(t))-I_{L}(t)\tau . \end{aligned}$$

For each strain to be in equilibrium, we require $\dot{I}_{H}=\dot{I}_{L}=0$. As long as $\beta _{H}\ne \beta _{L}$, this can never hold with strictly positive $I_{H}$ and $I_{L}$ when there is no intervention. This implies that the two strains can never co-exist. Instead, there are two possibilities. Either the Low strain will die out and the High strain will prevail, or both strains will die out. The Low strain dies out because the probability of infection in each encounter of a healthy individual with one infected with the Low strain is lower than in the healthy individual’s encounter with one infected with the High strain. As a result, the High strain spreads more quickly and eventually causes the Low strain to disappear. The High strain will prevail if $\beta _{H}>\tau$ and die out if $\beta _{H}<\tau$. Assumption (4) ensures that $\beta _{H}>\tau$ and so the High strain will prevail in equilibrium when there is no treatment, with $I_{H}^{*} =1-\frac{\tau }{\beta _{H}}$.

To determine the optimal treatment policy in the general model with treatment, the policymaker solves the current value Hamiltonian:

$$\begin{aligned} Q&=p(1-I_{H}-I_{L})-c(f_{H}I_{H}+f_{L}I_{L})\\&\quad +\lambda _{H}(\beta _{H}I_{H}(1-I_{H}-I_{L})-I_{H}(\tau +f_{H}\alpha )\\&\quad +\lambda _{L}(\beta _{L}I_{L}(1-I_{H}-I_{L})-I_{L}(\tau +f_{L}\alpha ). \end{aligned}$$

As the control variables $f_{H}$ and $f_{L}$ enter the Hamiltonian in a linear fashion, there is a bang-bang solution. The first-order conditions describing the optimal choice of treatment at a given point in time are

5 $$\begin{aligned} f_{H}\left\{ \begin{array} [c]{c} =0\\ \in [0,1]\\ =1 \end{array} \right\} \quad \text { if }\lambda _{H}\left\{ \begin{array} [c]{c} <\\ =\\ > \end{array} \right\} -\frac{c}{\alpha }, \end{aligned}$$

6 $$\begin{aligned} f_{L}\left\{ \begin{array} [c]{c} =0\\ \in [0,1]\\ =1 \end{array} \right\} \quad \text { if }\lambda _{L}\left\{ \begin{array} [c]{c} <\\ =\\ > \end{array} \right\} -\frac{c}{\alpha }, \end{aligned}$$

where we drop time subscripts in these and many subsequent expressions for ease of notation. These conditions imply that if $\lambda _{H}<-\frac{c}{\alpha }$ for example, then it is optimal to treat no one infected with the High strain. On the other hand, if $\lambda _{L}=-\frac{c}{\alpha }$, then the policymaker is indifferent between any level of treatment of the Low strain. These conditions can also be interpreted intuitively. Optimal treatment levels depend on the shadow price ($\lambda$) of having an additional infected individual in the population. This shadow price will differ for the two strains. Considering the *H* type as an example, the optimal value of $f_{H}$ will depend on how $\left| \lambda _{H}^{*}\right|$ compares to $\frac{c}{\alpha }$.^9^ The latter is the price of treating an individual divided by the probability that the treatment will be successful: it is the effective cost of treating the individual. It is constant for all infectees in the population. If the shadow price exceeds the effective cost of treatment, then all individuals are treated because the price of having them infected is greater than the price of treating them. On the other hand, if the shadow price is less than the effective cost, the price of treating an individual is greater than the price to social welfare of allowing this individual to remain infected and no one is treated. Finally, where the shadow price and effective cost are equal, the policymaker is indifferent between treating and not treating the individual and any level of treatment is optimal.

To complete the solution, the equations of motion for the two co-state variables are given below:

7 $$\begin{aligned} \dot{\lambda }_{H}&=\delta \lambda _{H}-\frac{\partial Q}{\partial I_{H} }\nonumber \\&=-p+cf_{H}-\lambda _{H}\left[ -\delta +\beta _{H}(1-I_{H}-I_{L})-\tau -\alpha f_{H}\right] \nonumber \\&\quad +\left( \lambda _{H}\beta _{H}I_{H}+\lambda _{L}\beta _{L}I_{L}\right) \end{aligned}$$

8 $$\begin{aligned} \dot{\lambda }_{L}&=\delta \lambda _{L}-\frac{\partial Q}{\partial I_{L} }\nonumber \\&=-p+cf_{L}-\lambda _{L}\left[ -\delta +\beta _{L}(1-I_{H}-I_{L})-\tau -\alpha f_{L}\right] \nonumber \\&\quad +\left( \lambda _{H}\beta _{H}I_{H}+\lambda _{L}\beta _{L}I_{L}\right) \end{aligned}$$

These demonstrate how the shadow prices change with prevalence levels. The reproduction ratio describes the number of new infections arising from one infection (Anderson and May 1991). If it is greater than one, the infection persists; otherwise, it dies out. In this model, the reproduction ratios of the High and Low strains are

$$\begin{aligned} R^{H}&=\frac{\beta _{H}}{\tau +\alpha f_{H}},\\ R^{L}&=\frac{\beta _{L}}{\tau +\alpha f_{L}}. \end{aligned}$$

As a result of Assumption (4), these reproduction ratios are greater than one even if everyone infected with either strain is treated, so that neither strain can be eradicated (except in the case with no treatment, when the High strain will eradicate the Low strain).

## Fixed points

In the analysis of dynamic models, of primary interest are the equilibria or fixed points of the model. In this section, we discuss the fixed points of the system and under which conditions they are attainable.

### Analysis of non-boundary fixed points

We propose that there are two types of equilibria: non-boundary fixed points and asymptotic fixed points. They are defined as follows.

#### Definition 1

A non-boundary fixed point (FP) is a solution $(f_{H}^{*},f_{L}^{*},I_{H}^{*},I_{L}^{*},\lambda _{H}^{*},\lambda _{L}^{*})$ satisfying Eqs. (2), (3), (5), (6), (7) and (8), as well as $\dot{I}_{H}=\dot{I} _{L}=\dot{\lambda }_{H}=\dot{\lambda }_{L}=\dot{f}_{H}=\dot{f}_{L}=0$.

#### Definition 2

An asymptotic fixed point (AFP) is a fixed point of the degenerate system in which one of $(I_{H},I_{L})$ equals zero and towards which there are paths with $I_{H}>0,I_{L}>0$ that converge asymptotically to this fixed point. We look for the treatment values $(f_{H}^{*},f_{L}^{*})$ in the vicinity of this fixed point that ensure this asymptotic convergence. On the boundary where one of the strains reaches zero prevalence (e.g. $I_{H}$), the co-state variable for that strain (e.g. $\lambda _{H}$) will be infinite, while the co-state variable of the other strain (e.g. $\lambda _{L}$) will be finite. In this sense, it is not a fixed point of the four variable system $(I_{H} ,I_{L},\lambda _{H},\lambda _{L})$.

The key difference between AFPs and FPs is that some variables at an AFP are not constant: they move towards a constant but only reach it in the limit. The following Lemma reduces the set of feasible non-boundary fixed points to three:

#### Lemma 1

At any fixed point, the proportion of $I_{H}$ treated must be greater than the proportion of $I_{L}$ treated: $f_{H}^{*}>f_{L}^{*}$.^10^ Therefore, only the following combinations of treatment at the fixed point are feasible:$\ (f_{H}^{*}=1,f_{L}^{*}=0);$ $(f_{H}^{*}=1,f_{L}^{*}\in (0,1));$ $(f_{H}^{*}\in (0,1),f_{L}^{*}=0)$. This implies that there are three potential non-boundary fixed points.

#### Proof

At a non-boundary fixed point, it must be the case that

$$\begin{aligned} I_{H}^{*}>0,\\ I_{L}^{*}&>0,\\ \dot{I}_{H}&=\dot{I}_{L}=0. \end{aligned}$$

This implies

$$\begin{aligned} 0= & {} \frac{\dot{I}_{H}}{I_{H}^{*}}=\beta _{H}(1-I_{H}^{*}-I_{L}^{*} )-\tau -\alpha f_{H}^{*},\\ 0= & {} \frac{\dot{I}_{L}}{I_{L}^{*}}=\beta _{L}(1-I_{H}^{*}-I_{L}^{*} )-\tau -\alpha f_{L}^{*}. \end{aligned}$$

Eliminating $$(1-I_{H}^{*}-I_{L}^{*})$$ yields

$$\begin{aligned} \beta _{L}\left( \tau +\alpha f_{H}^{*}\right) =\beta _{H}\left( \tau +\alpha f_{L}^{*}\right) , \end{aligned}$$

and hence

9 $$\begin{aligned} 1\ge f_{H}^{*}=\frac{\beta _{H}}{\beta _{L}}f_{L}^{*}+\frac{\tau }{\alpha }\left( \frac{\beta _{H}-\beta _{L}}{\beta _{L}}\right) >f_{L}^{*} \ge 0. \end{aligned}$$

$$\square $$

Since $f_{H}^{*}>0$ it follows that $\lambda _{H}^{*}\le -\frac{c}{\alpha }.$ Alternatively,

10 $$\begin{aligned} f_{L}^{*}=\frac{\beta _{L}}{\beta _{H}}\left[ f_{H}^{*}-\left( \frac{\beta _{H}-\beta _{L}}{\beta _{L}}\right) \frac{\tau }{\alpha }\right] . \end{aligned}$$

At a non-boundary fixed point $\dot{I}_{H}=0$ and $I_{H}$ $>0.$ From (2) it follows that the total prevalence of the disease is given by

11 $$\begin{aligned} I_{L}^{*}+I_{H}^{*}=1-\frac{\tau +\alpha f_{H}^{*}}{\beta _{H} }. \end{aligned}$$

Since $\dot{\lambda }_{H}=0$ it follows from (7) that

$$\begin{aligned} 0=-p+cf_{H}^{*}+\delta \lambda _{H}^{*}+\lambda _{H}^{*}\left[ \beta _{H}(1-I_{H}^{*}-I_{L}^{*})-\tau -\alpha f_{H}^{*}\right] +\lambda _{H}^{*}\beta _{H}I_{H}^{*}+\lambda _{L}^{*}\beta _{L} I_{L}^{*}. \end{aligned}$$

Eliminating $$I_{L}^{*}$$ yields

$$\begin{aligned} 0=-p+cf_{H}^{*}+\delta \lambda _{H}^{*}+\lambda _{H}^{*}\beta _{H} I_{H}^{*}+\lambda _{L}^{*}\beta _{L}\left( 1-\frac{\tau +\alpha f_{H}^{*}}{\beta _{H}}-I_{H}^{*}\right) , \end{aligned}$$

which can be written as

12 $$\begin{aligned} 0=-p+cf_{H}^{*}+\delta \lambda _{H}^{*}+\lambda _{L}^{*}\beta _{L}\left( 1-\frac{\tau +\alpha f_{H}^{*}}{\beta _{H}}\right) -\left( \lambda _{H}^{*}\beta _{H}-\lambda _{L}^{*}\beta _{L}\right) I_{H}^{*}. \end{aligned}$$

The above equation determines $I_{H}^{*}$ uniquely as a function of $f_{H}^{*}$, $\lambda _{H}^{*}$ and $\lambda _{L}^{*}$:

13 $$\begin{aligned} I_{H}^{*}=\frac{p+cf_{H}^{*}+\delta \lambda _{H}^{*}+\lambda _{L}^{*}\beta _{L}\left( 1-\frac{\tau +\alpha f_{H}^{*}}{\beta _{H}}\right) }{\left( \lambda _{L}^{*}\beta _{L}-\lambda _{H}^{*}\beta _{H}\right) }. \end{aligned}$$

At a non-boundary fixed point $\dot{\lambda }_{H}=\dot{\lambda }_{L}=0.$ Subtracting (8) from (7), using the fact that $\dot{I}_{H}=\dot{I}_{L}=0,$ and rearranging yields

14 $$\begin{aligned} f_{H}^{*}-f_{L}^{*}=-\frac{\delta }{c}\left( \lambda _{H}^{*} -\lambda _{L}^{*}\right) . \end{aligned}$$

Lemma 1 shows that $f_{H}^{*}-f_{L}^{*}>0$ at any non-boundary fixed point, so the above equation implies that $\lambda _{H}^{*}<\lambda _{L}^{*}.$

Following Wagener (2003), the parameter space can be split into different regimes that mandate which fixed points are feasible for which parameter constellations. We define the constant *K*, which will be used to create three regimes:

$$\begin{aligned} K=\frac{\beta _{H}-\beta _{L}}{\beta _{L}}\frac{\tau }{\alpha }. \end{aligned}$$

The value of this constant is increasing in $\beta _{H}$ and $\tau$ but decreasing in $\beta _{L}$ and $\alpha$. Intuitively, this implies that *K* will be high when the difference between the two infectivities is high and when the success rate of treatment is low but the natural rate of recovery is high. We will show that there are three regimes of feasibility: $K<1,K=1$ and $K>1$. We distinguish between two cases of fixed points, $f_{H}^{*} \in (0,1)$ and $f_{H}^{*}=1$.

#### Case 1: $f_{H}^{*}\in (0,1)$

We shall show that this case requires that $K=\left( \frac{\beta _{H} -\beta _{L}}{\beta _{L}}\right) \frac{\tau }{\alpha }<1$ and $K<\frac{\delta }{\alpha }.$ In this case $\lambda _{H}^{*}=-\frac{c}{\alpha }$ because $f_{H}^{*}$ takes an interior value. Hence from (14), $\lambda _{L}^{*}>-\frac{c}{\alpha }$and $f_{L}^{*}=0.$ From (9) and (14) it follows that this fixed point is characterised by the following values of the control variables and co-state variables:

$$\begin{aligned} f_{H}^{*}&=\frac{\beta _{H}}{\beta _{L}}f_{L}^{*}+\frac{\tau }{\alpha }\left( \frac{\beta _{H}-\beta _{L}}{\beta _{L}}\right) =K,\\ f_{L}^{*}&=0,\\ \lambda _{H}^{*}&=-\frac{c}{\alpha },\\ \lambda _{L}^{*}&=\frac{c}{\delta }\left( f_{H}^{*}-f_{L}^{*}\right) +\lambda _{H}^{*}=\frac{c}{\delta }\left[ K-\frac{\delta }{\alpha }\right] .\\ I_{H}^{*}&=\frac{p+cK-\frac{c}{\alpha }\delta +\frac{c}{\delta }\left[ K-\frac{\delta }{\alpha }\right] \beta _{L}\left( 1-\frac{\tau +\alpha K}{\beta _{H}}\right) }{\left( \frac{c}{\delta }\left[ K-\frac{\delta }{\alpha }\right] \beta _{L}+\frac{c}{\alpha }\beta _{H}\right) }>0 \end{aligned}$$

Thus, $f_{H}^{*}$ is decreasing in $\alpha$ but increasing in $\tau$. It is also increasing in $\beta _{H}$ because a more infectious strain requires more treatment but decreasing in $\beta _{L}$, as this implies a lower overall spread of the disease. Since $f_{H}^{*}<1$ the parameters must satisfy the following condition:

$$\begin{aligned} K<1. \end{aligned}$$

Economic logic requires that $\lambda _{L}^{*}<0,$ which yields the additional condition:

$$\begin{aligned} K<\frac{\delta }{\alpha }. \end{aligned}$$

Logic also requires that $I_{H}^{*}<1-\frac{\tau }{\beta _{L}}$, which yields the condition

$$\begin{aligned} \frac{p+cK-\frac{c}{\alpha }\delta +\frac{c}{\delta }\left[ K-\frac{\delta }{\alpha }\right] \beta _{L}\left( 1-\frac{\tau +\alpha K}{\beta _{H}}\right) }{\left( \frac{c}{\delta }\left[ K-\frac{\delta }{\alpha }\right] \beta _{L}+\frac{c}{\alpha }\beta _{H}\right) }<1-\frac{\tau }{\beta _{L}}. \end{aligned}$$

In practice, this places constraints on *c* and *p*, which are otherwise free parameters. The value of $I_{L}^{*}$ can be determined from the equation for total prevalence:

$$\begin{aligned} I_{H}^{*}+I_{L}^{*}=1-\frac{\tau }{\beta _{L}}. \end{aligned}$$

Total prevalence depends positively on $\beta _{L}$ and negatively on $\tau$; there is no dependence on $\beta _{H}$ as this enters through the choice of $f_{H}^{*}$. The success rate of treatment, $\alpha$, does not affect total prevalence because no one infected with the Low strain is treated; the dependence of the High strain on $\alpha$ enters, again, through the choice of $f_{H}^{*}$.

#### Case 2: $f_{H}^{*}=1$

Using (10), we find that in this case,

15 $$\begin{aligned} f_{L}^{*}=\frac{\beta _{L}}{\beta _{H}}\left[ f_{H}^{*}-\left( \frac{\beta _{H}-\beta _{L}}{\beta _{L}}\right) \frac{\tau }{\alpha }\right] =\frac{\beta _{L}}{\beta _{H}}\left[ 1-K\right] \end{aligned}$$

For this solution to be feasible, we must have $f_{L}^{*}\ge 0$ and hence

$$\begin{aligned} K=\left( \frac{\beta _{H}-\beta _{L}}{\beta _{L}}\right) \frac{\tau }{\alpha }\le 1. \end{aligned}$$

Therefore, two subcases of this fixed point can be distinguished, depending on whether $K=1$ or $K<1.$

**Case 2a** $f_{H}^{*}=1,K<1.$ Since $K<1$, this implies that $f_{L}^{*}=\frac{\beta _{L}}{\beta _{H} }\left[ 1-K\right] >0$. In particular, $f_{L}^{*}$ is increasing in $\beta _{L}$ but decreasing in $\beta _{H}$. There is interdependence between the two strains: the more infectious is the High strain, the less the Low strain is treated. However, the more infectious is the Low strain, the more it is treated. The optimal choice of $f_{L}^{*}$ also depends intuitively on $\alpha$ and $\tau$: the faster the rate of recovery, whether through treatment or naturally, the lower is the optimal treatment level. Since $f_{L}^{*}\in (0,1)$, it follows that $\lambda _{L}^{*}=-\frac{c}{\alpha }$ and the value of $\lambda _{H}^{*}$ is determined as follows:

$$\begin{aligned} \lambda _{H}^{*}&=\lambda _{L}^{*}-\frac{c}{\delta }\left( f_{H}^{*}-f_{L}^{*}\right) \\&=-\frac{c}{\alpha }-\frac{c}{\delta }\left( 1-\frac{\beta _{L}}{\beta _{H} }\left[ 1-K\right] \right) \\&<-\frac{c}{\alpha }. \end{aligned}$$

Thus, this fixed point is characterised by the following values of the control and co-state variables:

$$\begin{aligned} f_{H}^{*}&=1,\\ f_{L}^{*}&=\frac{\beta _{L}}{\beta _{H}}\left[ 1-K\right] \in (0,1),\\ \lambda _{L}^{*}&=-\frac{c}{\alpha },\\ \lambda _{H}^{*}&=-\frac{c}{\alpha }-\frac{c}{\delta }\left( 1-\frac{\beta _{L}}{\beta _{H}}\left[ 1-K\right] \right) <-\frac{c}{\alpha }. \end{aligned}$$

Inserting the above values of $f_{H}^{*},\lambda _{H}^{*}$ and $\lambda _{L}^{*}$ into (12) determines $I_{H}^{*}$ uniquely:

$$\begin{aligned} I_{H}^{*}=\frac{p-\frac{\delta c}{\alpha }+\frac{c\beta _{L}}{\beta _{H} }(1-K)-\frac{c\beta _{L}}{\alpha }\left( 1-\frac{\tau +\alpha }{\beta _{H} }\right) }{\frac{c}{\alpha }(\beta _{H}-\beta _{H})+\frac{c}{\delta }\beta _{H}\left( 1-\frac{\beta _{L}}{\beta _{H}}(1-K)\right) }>0. \end{aligned}$$

The value of $I_{L}^{*}$ can then be determined from (11):

$$\begin{aligned} I_{L}^{*}=1-\frac{\tau +\alpha }{\beta _{H}}-I_{H}^{*}, \end{aligned}$$

which is increasing in $\beta _{H}$ but decreasing in $\tau$ and $\alpha$. This is intuitive: the more infectious is the *H* type, the higher the prevalence of the disease in equilibrium; on the other hand, the more successful the treatment or the higher the rate of natural recovery, the lower the total prevalence in equilibrium. A point of interest is that $\beta _{L}$ does not affect total prevalence; this is because the level of $f_{L}^{*}$ is fixed and chosen based on the value of $\beta _{L}$. The dependence of $f_{L}^{*}$ on $\beta _{L}$ ensures that $\beta _{L}$ does not affect total prevalence. Note that we require $I_{H}^{*}$ and $I_{L}^{*}$ to be bounded by zero and one, so that an additional condition for the feasibility of this fixed point is

$$\begin{aligned} \frac{p-\frac{\delta c}{\alpha }+\frac{c\beta _{L}}{\beta _{H}}(1-K)-\frac{c\beta _{L}}{\alpha }\left( 1-\frac{\tau +\alpha }{\beta _{H}}\right) }{\frac{c}{\alpha }(\beta _{H}-\beta _{H})+\frac{c}{\delta }\beta _{H}\left( 1-\frac{\beta _{L}}{\beta _{H}}(1-K)\right) }<1-\frac{\tau +\alpha }{\beta _{H}}, \end{aligned}$$

which will place constraints on *p* and *c*.

**Case 2b** $f_{H}^{*}=1,$ $K=1.$ Equation (15) implies that in this case $f_{L}^{*}=0.$ Hence $\lambda _{H}^{*}\le -\frac{c}{\alpha }\le \lambda _{L}^{*}$ and

$$\begin{aligned} \lambda _{L}^{*}&=\lambda _{H}^{*}+\frac{c}{\delta }\left( f_{H}^{*}-f_{L}^{*}\right) \\&=\lambda _{H}^{*}+\frac{c}{\delta }. \end{aligned}$$

To ensure that $\lambda _{H}^{*}<-\frac{c}{\alpha }$and $\lambda _{L}^{*}<0$ we require that

$$\begin{aligned} -\left( \frac{c}{\alpha }+\frac{c}{\delta }\right) \le \lambda _{H}^{*}<-\max \left( \frac{c}{\alpha },\frac{c}{\delta }\right) . \end{aligned}$$

Thus, this fixed point is described as follows:

$$\begin{aligned} f_{H}^{*}&=1,\\ f_{L}^{*}&=0,\\ \lambda _{H}^{*}&:-\left( \frac{c}{\alpha \text { }}+\frac{c}{\delta }\right) \le \lambda _{H}^{*}<-\max \left( \frac{c}{\alpha \text { }} ,\frac{c}{\delta }\right) ,\\ \lambda _{L}^{*}&=\lambda _{H}^{*}+\frac{c}{\delta }. \end{aligned}$$

There is one degree of freedom. The shadow price $\lambda _{H}^{*}$ is indeterminate within the range shown. Eliminating $f_{H}^{*}$ and $\lambda _{L}^{*}$ from (12) determines $I_{H}^{*}$ as a function of $\lambda _{H}^{*}$. The value of $I_{L}^{*}$ can then be determined from (11). There is a continuum of feasible fixed points with total prevalence

$$\begin{aligned} I_{H}^{*}+I_{L}^{*}=1-\frac{\tau +\alpha }{\beta _{H}}. \end{aligned}$$

### Summary of non-boundary fixed points

This summary uses multiple asterisks (*), (**), and (***) to denote distinct types of fixed point. Note that feasibility requires that $\dot{I}_{H}=\dot{I}_{L}=0$ and $\dot{\lambda }_{H}=\dot{\lambda }_{L}=0.$

- If $K<1$ and $K\ge \frac{\delta }{\alpha }$ there is a unique non-boundary feasible fixed point $(I_{H}^{*},I_{L}^{*}).$ The control variables are $f_{H}^{*}=1$ and $f_{L}^{*}=\frac{\beta _{L}}{\beta _{H} }\left[ 1-K\right] .$
- If $$K<1$$ and $$K<\frac{\delta }{\alpha }$$ there are two feasible non-boundary fixed points: $$(I_{H}^{*},I_{L}^{*})$$ with the control variables $$f_{H}^{*}=1$$ and $$f_{L}^{*}=\frac{\beta _{L}}{\beta _{H} }\left[ 1-K\right] $$; and $$(I_{H}^{**},I_{L}^{**})$$ with control variables $$f_{H}^{**}=K$$ and $$f_{L}^{**}=0.$$
- If $$K=1\ $$the control variables satisfy $$f_{H}^{***}=1,f_{L}^{***}=0.$$ In this case, there is a continuum of feasible fixed points $$(I_{H}^{***},I_{L}^{***})$$ which lie on the line $$I_{H}^{***}+I_{L}^{***}=1-\frac{\tau +\alpha }{\beta _{H}}=1-\frac{\tau }{\beta _{L}}.$$

The above are the only feasible non-boundary fixed points. Note there are no feasible non-boundary fixed points if $K>1.$ These findings suggest the possibility of a Skiba case when $K<1$ and $K<\frac{\delta }{\alpha }$. Inserting control variables in (11) shows that fixed point (**) always has higher total infection than fixed point (*). This is intuitive as in the former, both treatment levels are lower. For ease of notation, we label fixed point (*) as $F^{low}$ (low total prevalence), fixed point (**) as $F^{high}$ (high total prevalence) and fixed point (***) as $F^{int}$ (intermediate total prevalence).

### Analysis of asymptotic fixed points

The asymptotic fixed points are defined such that one strain approaches eradication asymptotically, while the other prevails at a positive level. As a result, the behaviour of the system in the neighbourhood of the fixed point can be approximated by the behaviour of a one-infection system. This is because the behaviour of the system with small $I_{L}$ is very similar to the behaviour when $I_{L}=0$. Naturally, this holds for $I_{H}$ close to zero as well.

The behaviour of a one-infection system has been studied by Rowthorn (2006) and discussed in Sect. 2. Only boundary values for policy are optimal. This is because the co-state variable is a function of prevalence only in this type of problem. This implies that there must be a one-to-one mapping between prevalence and the co-state variable. However, the co-state variable changes with time. Therefore, a path cannot go back on itself in order to achieve two different values of the co-state variable for one level of prevalence. Interior values of policy require a path that goes back on itself (Rowthorn 2006). Therefore, interior values of policy cannot be optimal because they violate the assumption of the co-state variable being a function of prevalence.

To analyse asymptotic fixed points, we analyse a one-infection system and perturb it around this equilibrium point slightly. There are two possible categories of fixed points: those were $I_{H}$ tends asymptotically towards zero, and those where $I_{L}$ tends asymptotically towards zero.

#### $I_{H}=0$ boundary

First, suppose that $I_{H}=0.$ The system then reduces to the following maximisation problem:

$$\begin{aligned} \max V(I_{L}^{0})= {\int \nolimits _{0}^{\infty }} e^{-\delta t}\left[ p(1-I_{L}(t))-cf_{L}(t)I_{L}(t))\right] dt \end{aligned}$$

subject to

16 $$\begin{aligned} \dot{I}_{L}&=I_{L}(t)\left[ \beta _{L}(1-I_{L}(t))-\tau -\alpha f_{L}(t)\right] ,\nonumber \\ f_{L}(t)&\in [0,1],\nonumber \\ I_{L}(t)&\in [0,1],\nonumber \\ I_{L}(0)&=I_{L}^{0}>0,\nonumber \\ \beta _{L}&>\tau +\alpha . \end{aligned}$$

Optimal treatment is governed by the following bang-bang condition:

17 $$\begin{aligned} f_{L}\left\{ \begin{array} [c]{c} =0\\ \in [0,1]\\ =1 \end{array} \right\} \quad \text { if }\lambda _{L}\left\{ \begin{array} [c]{c} <\\ =\\ > \end{array} \right\} -\frac{c}{\alpha }. \end{aligned}$$

Finally, the co-state variable evolves according to the following function:

18 $$\begin{aligned} \dot{\lambda }_{L}&=\delta \lambda _{L}-\frac{\partial Q}{\partial I_{L} }\nonumber \\&=\delta \lambda _{L}+p+cf_{L}-\lambda _{L}\left[ \beta _{L}(1-I_{L} )-\tau -\alpha f_{L}\right] \nonumber \\&\quad +\lambda _{L}\beta _{L}I_{L}. \end{aligned}$$

At a Hamiltonian fixed point, $\dot{\lambda }_{L}=0$ and $\dot{I}_{L}=0$:

$$\begin{aligned} 0&=\left( \delta +\beta _{L}I_{L}\right) \lambda _{L}+p+cf_{L}\\ 0&=\beta _{L}(1-I_{L})-\tau -\alpha f_{L} \end{aligned}$$

If the fixed point is interior then $\lambda _{L}=-\frac{c}{\alpha }$ and we can solve the resulting equations to yield a unique interior fixed point $I_{L}^{int}.$ There are also fixed points with $f_{L}=0$ and $f_{L}=1.$ Let $I_{L}^{high},I_{L}^{low}$ be the corresponding levels of infection. It has been shown in the literature (Rowthorn 2006) that it is never optimal to go to $I_{L}^{int}$. Thus, the possible candidates for an optimum are $I_{L}^{high}$ and $I_{L}^{low}$. Let $A_{L}^{high}=(0,I_{L}^{high}),A_{L}^{low} =(0,I_{L}^{low}).$

First, note that the point $A_{L}^{low}$ cannot be reached even asymptotically from the interior. Consider any point $(x,I_{L}^{low}+y),$ where $x>0$:

19 $$\begin{aligned} \dot{I}_{H}&=I_{H}(t)\left[ \beta _{H}(1-I_{H}(t)-I_{L}(t))-\tau -\alpha f_{H}(t)\right] \nonumber \\&=x\left[ \beta _{H}(1-x-I_{L}^{low}-y)-\tau -\alpha f_{H}(t)\right] \nonumber \\&=x\left[ -\beta _{H}\left( x+y\right) +\beta _{H}(1-I_{L}^{low} )-\tau -\alpha f_{H}(t)\right] \end{aligned}$$

Recall that $\dot{I}_{L}=0$ implies that $\beta _{L}(1-I_{L}^{low})-\tau -\alpha =0$. Hence,

20 $$\begin{aligned} \dot{I}_{H}=x\left[ -\beta _{H}\left( x+y\right) +\left( \frac{\beta _{H} }{\beta _{L}}-1\right) (\tau +\alpha )+\alpha (1-f_{H}(t))\right] . \end{aligned}$$

For sufficiently small values of *x* and *y*,  and for any value $f_{H} (t)\in [0,1]$, the right hand side of this equation is positive. Hence, the prevalence of the High strain is increasing away from zero rather than decreasing towards zero, and $A_{L}^{low}$ cannot be reached even asymptotically from the interior.

What about the point $A_{L}^{high}?$ Consider any point $(x,I_{L}^{high}+y)$ where $x>0$:

21 $$\begin{aligned} \dot{I}_{H}&=I_{H}(t)\left[ \beta _{H}(1-I_{H}(t)-I_{L}(t))-\tau -\alpha f_{H}(t)\right] \nonumber \\&=x\left[ \beta _{LH}(1-x-I_{L}^{high}-y)-\tau -\alpha f_{H}(t)\right] \nonumber \\&=x\left[ -\beta _{H}\left( x+y\right) +\beta _{H}(1-I_{L}^{high} )-\tau -\alpha f_{H}(t)\right] \end{aligned}$$

As before, $\beta _{L}(1-I_{L}^{high})-\tau =0$, so that

22 $$\begin{aligned} \dot{I}_{H}=x\left[ -\beta _{H}\left( x+y\right) +\left( \frac{\beta _{H} }{\beta _{L}}-1\right) \tau -\alpha f_{H}(t)\right] . \end{aligned}$$

For sufficiently small values of *x* and *y*,  the right hand side is positive. In particular, for small *x* and *y* the right hand side is positive for all $f_{H}(t)\in [0,1]$ if

$$\begin{aligned} K=\left( \frac{\beta _{H}-\beta _{L}}{\beta _{L}}\right) \frac{\alpha }{\tau }>1. \end{aligned}$$

If this condition is satisfied, there is no path that converges to $A_{L}^{high}$ from the interior. On the other hand, for $f_{H}(t)=1$, the asymptotic fixed point $A_{L}^{high}$ is attainable if $K<1$. Thus, there is a feasible asymptotic fixed point with

$$\begin{aligned} f_{H}^{*}&=1,\\ f_{L}^{*}&=0,\\ I_{H}&\rightarrow 0,\\ I_{L}^{*}&=1-\frac{\tau }{\beta _{L}}. \end{aligned}$$

#### $I_{L}=0$ boundary

Suppose next that $I_{L}=0$. By symmetry, there are two potential categories of fixed points, with $f_{H}^{*}=0$ or $=1$. Let $I_{H}^{high},I_{H}^{low}$ be the corresponding levels of infection. When are these feasible?

Let $A_{H}^{high}=(0,I_{H}^{high}),$ and $A_{H}^{low}=(0,I_{H}^{low}).$ Note that the point $A_{L}^{low}$ can always be reached asymptotically from the interior. Consider any point $(I_{H}(t),I_{L}(t))=(I_{H}^{low}+x,y)$ where $y>0$. Suppose that $f_{H}^{*}=1$ and $I_{H}^{low}=1-\frac{\tau +\alpha }{\beta _{H}}$. In this case, the evolution of $I_{L}$ follows

23 $$\begin{aligned} \dot{I}_{L}=y\left[ -\beta _{L}(x+y)+\left( \frac{\beta _{L}}{\beta _{H}}-1\right) \tau +\alpha \frac{\beta _{L}}{\beta _{H}}-\alpha f_{L}(t)\right] . \end{aligned}$$

The right-hand side of this equation is negative for small *x* and *y* when $f_{L}>\frac{\beta _{L}}{\beta _{H}}>0$. Since an interior value of $f_{L}$ is suboptimal, this implies there is one final asymptotic fixed point, which is feasible for all *K*:

$$\begin{aligned} f_{L}^{*}&=1,\\ f_{H}^{*}&=1,\\ I_{L}&\rightarrow 0,\\ I_{H}^{*}&=1-\frac{\tau +\alpha }{\beta _{H}}. \end{aligned}$$

What about $A_{H}^{high}$ with $f_{H}^{*}=0$? Consider any point $(I_{H}(t),I_{L}(t))=(I_{H}^{high}+x,y)$ where $y>0.$ The evolution of $I_{L}$ is governed by the following equation:

24 $$\begin{aligned} \dot{I}_{L}=y\left[ -\beta _{L}(x+y)+\left( \frac{\beta _{L}}{\beta _{H}}-1\right) \tau -\alpha f_{L}(t)\right] . \end{aligned}$$

The right-hand side is negative for all values of $f_{L}(t)\in [0,1].$ Since interior values of $f_{L}$ can be ruled out by Rowthorn (2006), this asymptotic fixed points can be reached for all values of *K* by setting $f_{L}=0$ or $f_{L}=1$:

$$\begin{aligned} f_{L}^{*}&\in \{0,1\},\\ f_{H}^{*}&=0,\\ I_{L}&\rightarrow 0,\\ I_{H}^{*}&=1-\frac{\tau }{\beta _{H}}. \end{aligned}$$

Thus, when $K>1$, the only feasible candidates for asymptotic equilibria are those with $I_{L}\rightarrow 0$.

### Summary of asymptotic fixed points

If $K<1$, there are three asymptotic fixed points:

- $$A_{L}^{high}$$, with $$f_{H}^{*}=1$$, $$f_{L}^{*}=0$$ and $$I_{H}\rightarrow 0,$$
- $$A_{H}^{high}$$ with $$f_{L}^{*}\in \{0,1\}$$, $$f_{H}^{*}=0$$ and $$I_{L}\rightarrow 0,$$
- $$A_{H}^{low}$$ with $$f_{L}^{*}=1$$, $$f_{H}^{*}=1$$ and $$I_{L}\rightarrow 0.$$

If $K>1$ there are no interior points that satisfy the Hamiltonian conditions. Moreover, the boundary points with $I_{H}=0$ cannot be reached. In this case, the only feasible candidates for an optimum are boundary points with $I_{L}=0.$ In particular, there are three possible cases with the following control variables:

- $$A_{H}^{high}$$ with $$f_{L}^{*}\in \{0,1\}$$, $$f_{H}^{*}=0$$ and $$I_{L}\rightarrow 0,$$
- $$A_{H}^{low}$$ with $$f_{L}^{*}=1$$, $$f_{H}^{*}=1$$ and $$I_{L}\rightarrow 0,$$
- Skiba case. The optimum path goes to either $A_{H}^{low}$ or $A_{H}^{high}$ depending on the starting point.

The central conclusion when $K>1$ is that optimal policy involves asymptotic elimination of the low infectivity strain. The possibility of the Skiba case indicates that it is not enough simply to compare fixed points—the initial prevalence of the disease will affect which fixed point is optimal.

However, the following Proposition shows that asymptotic eradication of both strains of the disease is never possible:

#### Proposition 1

Both variants of the disease cannot be simultaneously eradicated even asymptotically in equilibrium if

$$\begin{aligned} \frac{\tau +\alpha }{\beta _{H}}<1,\\ \frac{\tau +\alpha }{\beta _{L}}&<1, \end{aligned}$$

which hold as a consequence of Assumption (4).

#### Proof

In Appendix B. $$\square $$

This proposition shows that even if both AFPs are feasible, asymptotic eradication of the disease is not possible and at least one strain always prevails.

### Extensions to the model

In reality, strains do vary in factors such as drug resistance and the cost of treatment. In the present model, this could be modelled by varying the parameters $\alpha ,\tau$ and *c* by strain.

Suppose that the natural recovery rate of strain *H* is $\tau _{H}$ and that of strain *L* is $\tau _{L}$. Similarly, the success rate of treatment of the High strain is $\alpha _{H}$ and of the Low strain is $\alpha _{L}$. We can make some tentative claims about what would happen to the equilibria of the model. First, it should be noticed that coexistence of the two strains without intervention is now possible. The two strains evolve according to the following differential equations:

$$\begin{aligned} \frac{\dot{I}_{H}}{I_{H}}&=(1-I_{H}-I_{L})\beta _{H}-\tau _{H}-\alpha _{H}f_{H},\\ \frac{\dot{I}_{L}}{I_{L}}&=(1-I_{H}-I_{L})\beta _{L}-\tau _{L}-\alpha _{L}f_{L}. \end{aligned}$$

When there is no intervention, the strains can co-exist if

$$\begin{aligned} \frac{\tau _{H}}{\beta _{H}}=\frac{\tau _{L}}{\beta _{L}}. \end{aligned}$$

Intuitively, this condition requires that the rate of infection and rate of recovery of the two strains are proportional. Thus, the High strain spreads its infection more quickly but also returns healthy individuals back into the population more quickly, so that the Low strain can survive. Next, we can analyse what would happen to the non-boundary fixed points. Lemma 1 states that only those fixed points where the High strain is treated more are feasible. In fact, if $\alpha _{H}$ is higher than $\alpha _{L}$, and $\tau _{H}$ is higher than $\tau _{L}$, this result could be reversed. A non-boundary fixed point requires

$$\begin{aligned} \frac{\tau _{H}+\alpha _{H}f_{H}}{\beta _{H}}=\frac{\tau _{L}+\alpha _{L}f_{L} }{\beta _{L}}, \end{aligned}$$

so that the relationship between the two treatment levels is

$$\begin{aligned} f_{L}=\frac{\alpha _{H}\beta _{L}}{\alpha _{L}\beta _{H}}f_{H}+\frac{\beta _{L} \tau _{H}-\beta _{H}\tau _{L}}{\alpha _{L}\beta _{H}}. \end{aligned}$$

Thus, depending on the values of $\alpha _{H},\alpha _{L},\tau _{H}$ and $\tau _{L}$, $f_{L}$ could be higher than $f_{H}$. This makes intuitive sense, as higher values of $\alpha _{H}$ and $\tau _{H}$ make the High strain comparatively easier to contain, so that this may outweigh the fact that a higher transmission probability makes the High strain more difficult to contain. Finally, we can consider the implications for the asymptotic fixed points. Here, also, the main result may be reversed: namely that the only feasible equilibrium is one where the Low strain is asymptotically eradicated. This is easy to see by rewriting Eqs. (19) and (21) allowing for differences in $\alpha$ and $\tau$ by strain, and performing a similar exercise for asymptotic eradication of the Low strain. When $\alpha _{H}$ is much higher than $\alpha _{L}$, and similarly $\tau _{H}$ is much higher than $\tau _{L}$, then it may be that the only feasible equilibrium is asymptotic eradication of the High strain. This is because the low success rate of treatment of the Low strain, and its low natural rate of recovery, make it impossible to eradicate, even asymptotically.

Varying cost by strain would not affect prevalence or the fixed points that can be attained by the policymaker, but would affect the choice of equilibrium to converge to. This is ultimately a numerical question. It seems intuitive to assume that $c_{H}>c_{L}$: it is more expensive to treat those infected with the High strain. The effect on the optimal equilibrium would depend how these values compare to the homogeneous cost parameter *c*. It could be that the overall cost goes down for a given level of treatment (such as if $c_{H}$ is slightly higher than *c*, but $c_{L}$ is much lower than *c*), in which case the policymaker would be more likely to pick an equilibrium where more people are treated, such as $F^{low}$.

### Feasibility

#### Summary of regimes of feasible equilibria

In this section we plot the feasible equilibria for each regime of *K*. Recall that if $K<1$, the set of feasible equilibria is $Z_{K<1}=\{F^{low} ,F^{high},A_{L}^{high},A_{H}^{high},A_{H}^{low}\}$. If $K=1$, the set of feasible equilibria is $Z_{K=1}=\{F^{int},A_{H}^{high},A_{H}^{low}\}$. If $K>1$, the set of feasible equilibria is $Z_{K>1}=\{A_{H}^{high},A_{H} ^{low}\}$. As *K* increases, the set of feasible equilibria falls: there are fewer ways in which the disease can be contained. This is because a higher *K* occurs when treatment is ineffective or when the strains differ substantially in their infectivities, making it difficult to control both of them.

As *K* increases, the minimum attainable equilibrium prevalence of $I_{H}$ rises. This makes intuitive sense, as *K* is increasing in $\beta _{H}$ and in the difference between $\beta _{H}$ and $\beta _{L}$. Where this difference is very large, $K>1$ and the only feasible equilibrium is the AFP where $I_{L}$ tends asymptotically towards zero and $I_{H}$ prevails at a positive level. In contrast, when $\beta _{H}$ is low, $K<1$ and one can attain equilibria such as $F^{low}$, where the equilibrium prevalence of $I_{H}$ can be close to zero.

**Fig. 1:**
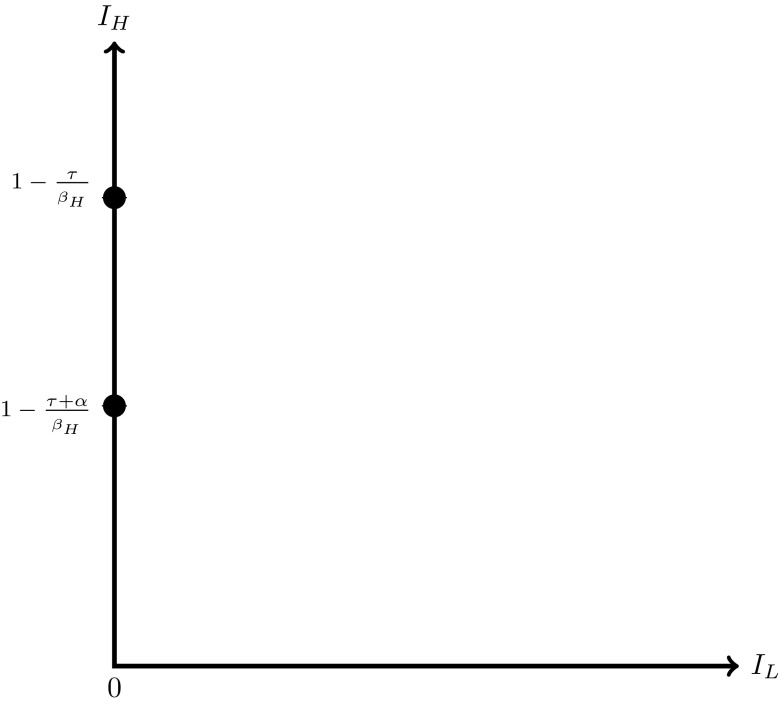
The set of feasible equilibria when $K>1$. The *full circles* denote the two equilibria $A_{H}^{high}$ and $A_{H}^{low}$

**Fig. 2:**
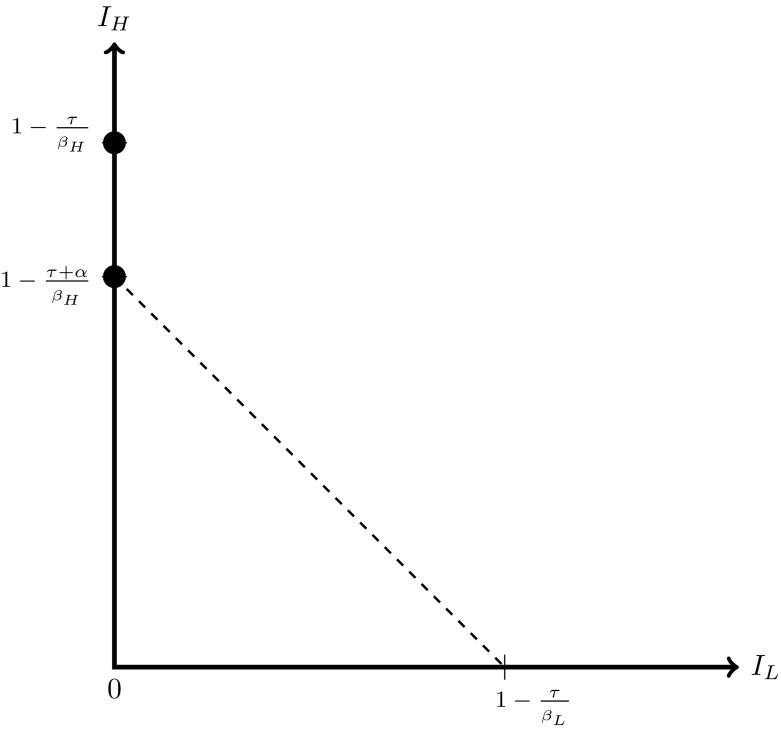
The set of feasible equilibria when $K=1$. The *full circles* show the equilibria $A_{H}^{high}$ and $A_{H}^{low}$. The *dashed line* denotes the continuum of equilibria of type $F^{int}$

**Fig. 3:**
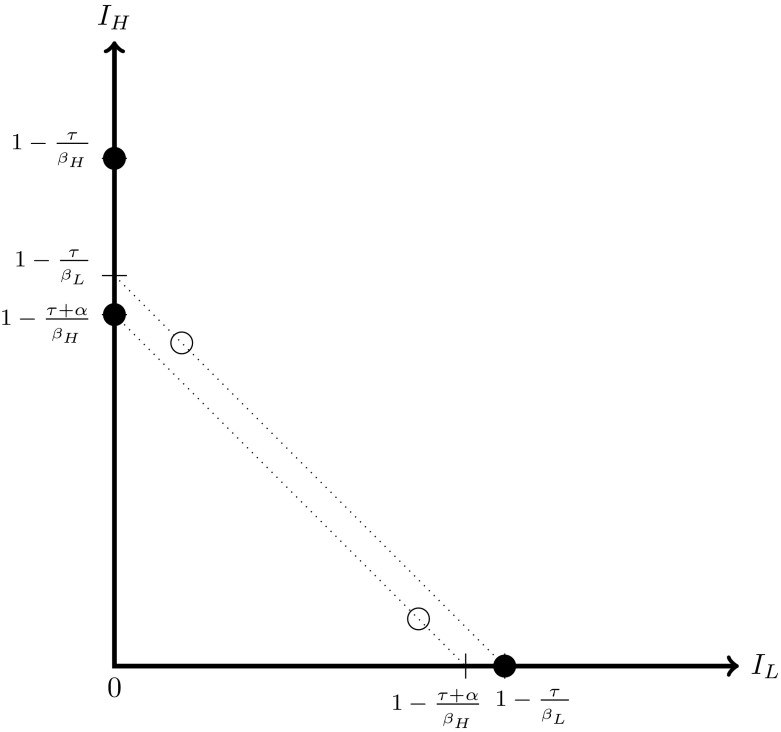
The set of feasible equilibria when $K<1$. The *full circles* denote the equilibria $A_{L}^{high}$ (on the x-axis) $,A_{H}^{high}$ and $A_{H} ^{low}$ (on the y-axis). The *empty circles* denote the feasible equilibria $F^{low}$ and $F^{high}$ for this set of parameters, with the latter having higher total prevalence

The set of feasible equilibria can be depicted graphically. Figure 1 shows the set of feasible equilibria when $K>1$. The feasible set when $K=1$ is shown in Fig. 2. Figure 3 shows the set of feasible equilibria when $K<1$.^11^ The graphs clearly illustrate the rising number of feasible equilibria as *K* falls.

#### An intuitive explanation and some examples

It is useful to introduce some parameter values in order to better understand the policy implications of these results. There are, in total, five individual fixed points: three non-boundary fixed points (one of which is in fact a continuum of feasible fixed points with the same total prevalence across the two strains) and two asymptotic fixed points. Feasibility depends on the value of $K=\left( \frac{\beta _{H}-\beta _{L}}{\beta _{L}}\right) \frac{\tau }{\alpha }$. In order for the interior fixed points to exist, we require $K\le 1$. If $K<1$ and $K<\frac{\delta }{\alpha }$, then two non-boundary fixed points exist, which suggest the existence of a Skiba line. A further consideration is whether the assumption $\beta _{H}>\beta _{L}>\tau +\alpha$ is satisfied, which ensures that the disease cannot be eradicated, even asymptotically.

Generally, the existence of a non-boundary fixed point with positive equilibrium prevalence of the disease requires the following conditions, where (a) to (c) ensure the existence of the non-boundary fixed points and (d) ensures that at this non-boundary fixed point, the disease is not eradicated.

- The relative difference between the infectivities, $\frac{\beta _{H}-\beta _{L}}{\beta _{L}}$, is not too high.
- The natural recovery rate is not too high relative to the success rate of treatment: $\frac{\tau }{\alpha }$ is not too high.
- The future is not discounted too highly: $\delta$ is high enough.
- The infectivity probabilities, $\beta _{H}$ and $\beta _{L}$, are high relative to the recovery probabilities, $\tau$ and $\alpha$

We can interpret these conditions in an intuitive way. In order for both strains of the disease to co-exist in equilibrium, we require that their infectivities are not too different. This is because if the High strain is much more infectious, then individuals will be much more susceptible to it relative to the Low strain, so that the Low strain will eventually die out (asymptotically). On the other hand, if the infection probabilities are similar but low, relative to the recovery probabilities, then both strains will die out, because the rate of reinfection is not high enough to ensure the survival of the disease. The magnitude of the natural recovery rate relative to the success rate of treatment is important because a high natural recovery rate will likely lead to the elimination of both strains of the disease, while a low value of $\alpha$ will imply that treatment is ineffective (and a particular level of treatment is required to ensure that the disease survives at a positive, constant level in equilibrium). Finally, the condition on the discount rate arises because economic logic requires $\lambda _{L}^{*}<0$ in the non-boundary fixed point $F^{high}$; otherwise, the marginal social value of an additional infected person would be positive. Intuitively, not discounting the future too highly ensures that the impact of an additional infected person is felt for a longer period of time, hence ensuring that the marginal social impact of this infected person is negative at the fixed point.

Let us consider two examples that illustrate these points. First, suppose that $\beta _{H}=0.9$, $\beta _{L}=0.1$: the High strain is very infectious, relative to the Low strain. In order for $K<1$, we require $\tau <\frac{\alpha }{8}$: the treatment needs to be very effective, relative to the natural rate of recovery. In addition, we require $\tau +\alpha <0.1$, in order for the disease to not be eradicated. One possible set of values for $(\tau ,\alpha )$ is (0.004, 0.04). Thus, in the example of a disease where the High strain is very infectious, the only way that both strains can co-exist in a non-boundary fixed point is when both the natural rate of recovery and the success rate of treatment are extremely low. Note that this example also requires a value of $\delta >0.8$, which is reasonable, given that the economics literature usually assumes a discount rate of 0.95 or 0.99 (the latter is implied by an annual interest rate of 4%). If natural recovery is somewhat higher, e.g. if $\tau =\alpha =0.04$, then we will end up in the situation where the Low strain is asymptotically eradicated, and the High strain prevails in equilibrium. If either the natural recovery rate or the treatment success are high, e.g. if $\alpha =0.2$, even if $\tau =0.004$, then both strains will be (asymptotically) eradicated.

Second, suppose that we have a very effective form of treatment with $\alpha =0.9$, but individuals naturally recover from the disease only rarely, so that $\tau =0.1$. In this case, the disease will be eradicated, because the condition $\beta _{H}>\beta _{L}>\tau +\alpha$ can never be satisfied. Suppose that instead we have a disease where $\alpha =0.8$ and $\tau =0.1$. Here, we require $\beta _{H}>\beta _{L}>0.9$, so that infection is almost guaranteed when a susceptible individual meets an infected individual of either strain. One combination of values of $\left( \beta _{H},\beta _{L}\right)$ that ensures coexistence of both strains in equilibrium is (0.99, 0.95). This set of parameters requires $\delta >0.004$, which is easily satisfied.

The general lesson from these examples is that where treatment is very successful, the disease needs to be highly infectious in order to prevail. An interesting finding is that the rate of natural recovery relative to the success of treatment is also important: coexistence of both strains is more likely when natural recovery is low, but the treatment is very successful. Finally, coexistence of both strains is more likely when they are similar in their infectivities. This makes sense intuitively, as the bigger the difference, the more likely that the infectivity of the High strain will cause the Low strain to die out.

#### Feasible policies along the path to the fixed points

Another area where restrictions on feasibility may apply is the choice of treatment policy along the path to the non-boundary fixed points. Policies along the path towards the fixed points will always be boundary policies (i.e. 0 or 1), as these are Most Rapid Approach Paths. We take each of the boundary policies in turn and examine the feasibility conditions required for $I_{H}$ and $I_{L}$ to converge to their equilibrium values if this is the chosen policy. Different feasibility constraints will exist depending on whether the system begins *below* or *above* each equilibrium; below means beginning at an initial level $I_{H}^{0}+I_{L}^{0}<I_{H}^{*}+I_{L}^{*}$, which requires $I_{H}$ and $I_{L}$ to *increase* to their equilibrium values, while beginning above implies an initial prevalence $I_{H}^{0}+I_{L}^{0}>I_{H}^{*}+I_{L}^{*}$, which requires $I_{H}$ and $I_{L}$ to *decrease* to their equilibrium values. Letting $P^{ab}$ denote the policy $f_{H}=a$, $f_{L}=b$, the feasibility restrictions are summarised in Table 1. Derivations can be found in Appendix C.

**Table 1:** Feasible policies along the path

	$\dot{I}_{H},\dot{I}_{L}>0$	$\dot{I}_{H},\dot{I}_{L}<0$
$P^{00}$	$1-\frac{\tau }{\beta _{L}}>I_{H}+I_{L}$	$1-\frac{\tau }{\beta _{H}}<I_{H}+I_{L}$
$P^{10}$	$1-\frac{\tau }{\beta _{L}}>I_{H}+I_{L}$	$1-\frac{\tau +\alpha }{\beta _{H}}<I_{H}+I_{L}$
$P^{11}$	$1-\frac{\tau +\alpha }{\beta _{L}}>I_{H}+I_{L}$	$1-\frac{\tau +\alpha }{\beta _{H}}<I_{H}+I_{L}$
$P^{01}$	$1-\frac{\tau +\alpha }{\beta _{L}}>I_{H}+I_{L}$	$1-\frac{\tau }{\beta _{H}}<I_{H}+I_{L}$

**Fig. 4:**
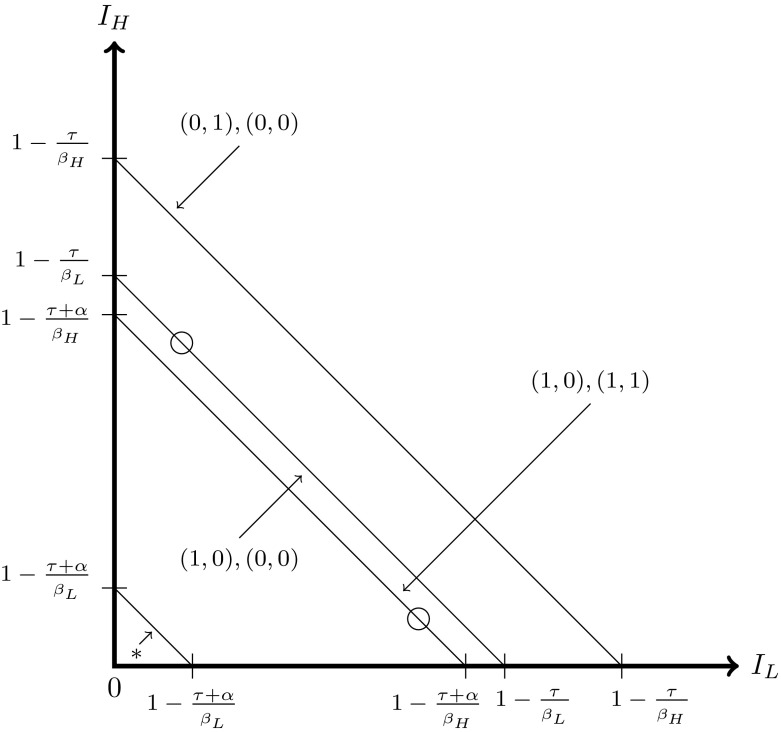
Feasible policies depicted for the case when $K<1$, where the *arrows* denote the range where the policy $(f_{H},f_{L})$ is feasible. At $*$, the policies are (0, 1) and (1, 1). The *empty circles* denote the feasible equilibria $F^{high}$ and $F^{low}$ for this set of parameters

The table is interpreted as follows. If the system begins below $I_{H}^{*}+I_{L}^{*}$, $I_{H}$ and $I_{L}$ need to increase. Not all policies can achieve this at all times: the range where the policy $f_{H}=0,f_{L}=0$ will allow $I_{H}$ and $I_{L}$ to increase is given by the top left entry in the table. There is an *upper bound* on $I_{H}+I_{L}$; if this condition is not satisfied, then setting $f_{H}=0,f_{L}=0$ will cause the system to move away from an equilibrium. The conditions in the table are more clearly shown in graphical form in Fig. 4.

The message of this figure is that when one starts below an equilibrium, feasible policies to reach the equilibrium usually involve treating no one or just one strain. This allows total prevalence to increase. On the other hand, when one starts above an equilibrium, feasible policies that ensure the equilibrium is reached treat at least one group, in order to ensure that prevalence falls. Thus, $F^{high}$ and $F^{low}$ can be reached from above with the policies $(f_{H},f_{L})=(1,0)$ and (1, 1), while they can be reached from below with the policies (1, 0) and (0, 0).

### Summary of analytical results

Table 2 summarises the analytical results—the feasible fixed points, their associated optimal policies and for which values of *K* they are attainable.

**Table 2:** Summary of analytical results

Equilibrium	$f_{H}^{*}$	$f_{L}^{*}$	Feasible
$F^{high}$	$\frac{(\beta _{H}-\beta _{L})}{\beta _{L}}\frac{\tau }{\alpha }$	0	$K<1,K<\frac{\delta }{\alpha }$
$F^{low}$	1	$1-\frac{(\beta _{H}-\beta _{L})}{\beta _{H}}\frac{\tau +\alpha }{\alpha }$	$K<1$
$F^{int}$	1	0	$K=1$
$A_{L}^{high}$	1	0	$K<1$
$A_{H}^{high}$	0	$\in \{0,1\}$	All *K*
$A_{H}^{low}$	1	1	All *K*

## Simulations

In this section, we provide examples of optimal policy under various parameter combinations. When $K>1$, the candidate equilibria are $A_{H}^{high}$ and $A_{H}^{low}$. Simulations can tell us, under particular parameter values, which equilibrium it is optimal to go to, and what is the best way to get there. We explore the case when $K>1$ in the first part of this section. Next, we consider the situation when $K<1$. In this case, there is a wide set of feasible equilibria, and it is not possible to simulate an optimal choice of equilibrium because the derivations of optimal policy are only correct in the neighbourhood of each equilibrium. Instead, we suppose that it is optimal to go to the non-boundary fixed point $F^{low}$, and simulate optimal policy towards this point. We find that, in all the simulations that we estimate, optimal policy involves a spiral. We argue that this suggests that the interior fixed points are unlikely to ever be an optimal target for the policymaker.^12^

### Optimal policy when $K>1$

The values used for the parameters in the simulations are given in Table 3.

**Table 3:** Parameter values for K > 1

Parameter	Value
$\beta _{H}$	0.95
$\beta _{L}$	0.4
$\tau$	0.15
$\alpha$	0.2
*p*	1
$\delta$	0.111

Some relevant concepts ought to be explained at this stage. First, we examine both optimal fixed and optimal variable policy. Optimal fixed policy compares the social welfare from choosing various policies at the beginning and not changing them until equilibrium. Optimal variable policy uses the Hamiltonian optimality conditions (5) and (6) to evaluate optimal policy at each time increment, using a fourth-order Runge-Kutta procedure. Paths with optimal variable policy are described as Hamiltonian paths because they satisfy these Hamiltonian optimality conditions. In the case of variable policy, we examine both forward and backward paths. This means that, starting from an initial prevalence, we simulate the system both towards and away from the fixed point. This is carried out by setting time increments equal to $+1$ and $-1$ respectively. As a result, it is possible to describe a longer optimal path than if we were to only simulate the system towards equilibrium.

#### Fixed policy

In this first set of simulations, we assume that policy is fixed along the entire path to equilibrium. This is a special case that can guide intuition, as policy may not always have the benefit of full flexibility. We assume that the policymaker chooses $f_{L}^{*}=1$, and compare social welfare *V* when $f_{H}^{*}=1$ or $f_{H}^{*}=0$. When the system begins, the policymaker chooses whether to treat everyone or no one infected with the High strain. The optimal policy is the choice of $f_{H}^{*}$ that gives the highest value of social welfare.^13^ The simulations set $t=90$. Each time increment can be interpreted as one day, which implies that the results simulate an infection evolving over 90 days. The initial prevalence of the Low strain is set to 0.1.

Results are shown in Table 4. Optimal policy depends on costs. Where costs are low, it is optimal to treat everyone infected with the High strain. As costs rise, it becomes optimal to treat no one with the High strain. There is a unique optimal policy in regions *I* and *III*; in region *II*, optimal policy depends on the initial prevalence of the High strain. If the High strain is very prevalent, it is optimal to treat no one. If it is not, it is optimal to treat everyone. This strongly suggests that there exists a value of $I_{H}^{0}$ that is a Skiba point in Region *II*. By varying $I_{H}^{0}$ by small increments in region *II* and evaluating social welfare at each initial value for the two candidate policies, we find that a Skiba point exists when $I_{H}^{0}=0.7125$. At this point, the social welfare from selecting $f_{H}^{*}=1$ or $f_{H}^{*}=0$ is equal.^14^

**Table 4:** Optimal fixed policy as cost varies

Region	*c*	$f_{H}^{*}$	$f_{L}^{*}$
*I* (low costs)	$c<0.2875$	1	1
*II* (intermediate costs)	$0.2875\le c\le 0.3006$	0 or 1, depending on $I_{H}^{0}$	1
*III* (high costs)	$c>0.3006$	0	1

#### Variable policy

In this set of simulations we allow policy to vary optimally over time. We look for strictly Hamiltonian paths; that is, we look for paths that are optimal because they satisfy the first-order conditions on $\lambda _{H}$ and $\lambda _{L}$. For example, the policy $(f_{H}=1,f_{L}=1$) is Hamiltonian at a particular point in time $\bar{t}$ if $\left| \lambda _{H}\right| ,\left| \lambda _{L}\right| >\frac{c}{\alpha }$ at $(I_{H}(\bar{t}),I_{L}(\bar{t}))$. In order to evaluate the Hamiltonian conditions, the co-state variables are needed at each point in time. These can be calculated using the facts that

$$\begin{aligned} \lambda _{H}(t)&=\frac{\partial V(I_{H}(t),I_{L}(t))}{\partial I_{H}(t)},\\ \lambda _{L}(t)&=\frac{\partial V(I_{H}(t),I_{L}(t))}{\partial I_{L}(t)}. \end{aligned}$$

These partial derivatives can be approximated by perturbing the infection levels slightly. Thus, for initial infection levels $I_{H}^{0}$ and $I_{L} ^{0}$, the initial values of the co-state variables are

25 $$\begin{aligned} \lambda _{H}^{0}&\approx \frac{V(I_{H}^{0}+\Delta ,I_{L}^{0})-V(I_{H} ^{0},I_{L}^{0})}{\Delta }, \end{aligned}$$

26 $$\begin{aligned} \lambda _{L}^{0}&\approx \frac{V(I_{H}^{0},I_{L}^{0}+\Delta )-V(I_{H} ^{0},I_{L}^{0})}{\Delta }, \end{aligned}$$

for small $\Delta$. In these simulations we set $\Delta =0.001$. For a given initial infection level, we find the initial values of the co-state variables $\lambda _{H}^{0}$ and $\lambda _{L}^{0}$ using (25) and (26), and find the corresponding optimal initial policy. We then allow the system to evolve, checking for optimal policy at each increment of time by re-evaluating $\lambda _{H}(t)$ and $\lambda _{L}(t)$ using the equations for $\dot{\lambda }_{H}$ and $\dot{\lambda }_{L}$ that were derived in Sect. 3. We also simulate the paths backwards, starting at the initial point $(I_{H}^{0},I_{L}^{0})$ but moving away from equilibrium.^15^ Together, the forward and backward paths give a fairly complete picture of optimal policy over a period of length 2*t*, with $(I_{H}^{0},I_{L}^{0})$ in the middle. In this set of simulations we set $t=30$, so that overall we examine the behaviour of the infection over 60 days. We assume costs are $c=0.4$ and set initial prevalence at $(I_{H}^{0},I_{L}^{0})=(0.6316,2*10^{-10})$.

**Fig. 5:**
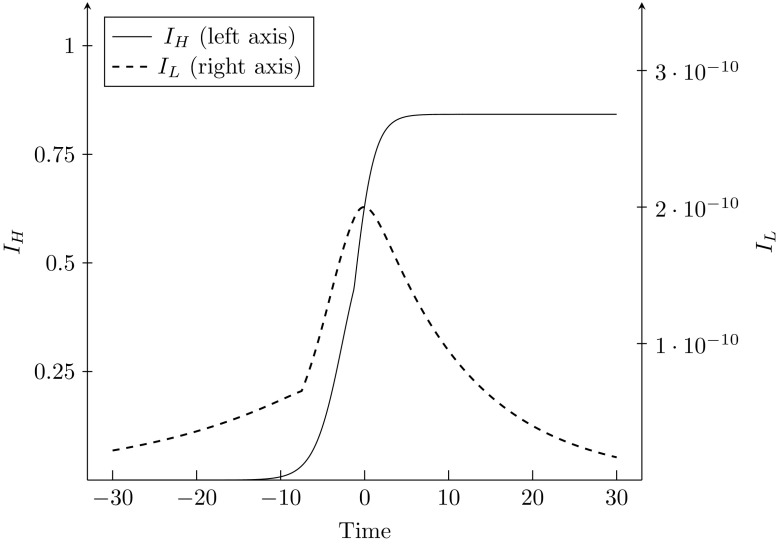
Evolution of prevalence along the path towards equilibrium $$A_{L}^{high}$$, with $$I_{H}^{0}=0.6316,$$ $$I_{L}^{0}=2*10^{-10}$$ and $$c=0.4 $$. Time >0 is the forward path, while time <0 is the backward path

We find that the system tends towards the equilibrium where $f_{H}^{*}=0$, so that $I_{H}^{*}=0.8421=1-\frac{\tau }{\beta _{H}}$ and $I_{L}^{*}\rightarrow 0.$ This is consistent with the findings in Table 4, as $c=0.4$ falls into Region $$III\ $$of costs. The evolution of the two strains is depicted in Fig. 5. This figure shows both the forward and backward paths. We find that prevalence of $I_{H}$ rises from a low initial point at first exponentially, and then roughly logarithmically until reaching the fixed point. Attainment of equilibrium takes approximately 15 days. The evolution of $I_{L}$ takes a slightly different path. Prevalence first increases, and then decreases towards zero. This is because as $I_{H}$ increases, there are fewer susceptibles that can become infected with the Low strain, so that the Low strain can be asymptotically eradicated.

**Fig. 6:**
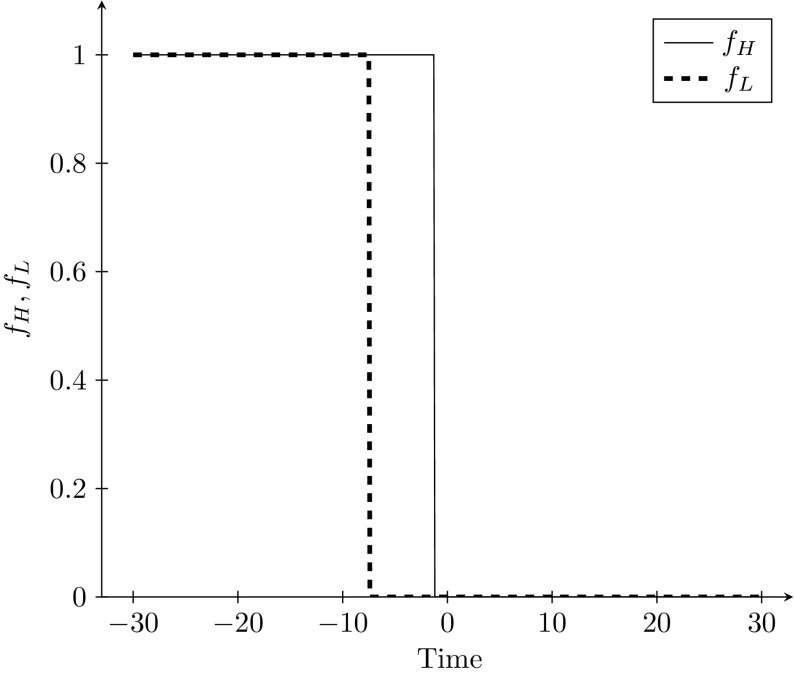
Optimal policy along the path towards $A_{L}^{high}$, with $I_{H} ^{0}=0.6316,$ $I_{L}^{0}=2*10^{-10}$ and $c=0.4$. Time $>0$ is the forward path, while time $<0$ is the backward path

When examining the sequence of optimal policy, we find that optimal policy exhibits switch points. This is depicted in Fig. 6. The treatment of the High strain switches from one to zero just before the start of the forward path, while the treatment of the Low strain switches from one to zero earlier.

Thus, optimal policy in this particular example has three phases:

$$\begin{aligned} f_{H}^{*}&=1,f_{L}^{*}=1,\\ f_{H}^{*}&=1,f_{L}^{*}=0,\\ f_{H}^{*}&=0,f_{L}^{*}=0. \end{aligned}$$

Thus, when policy is variable, it is always optimal to set $f_{H}^{*}=0$ in equilibrium when $c=0.4$. However, policy does not begin at this level: it switches from one to zero when the marginal cost of an additional infected individual in the population is low, relative to the cost of treating him. In addition, the asymptotic eradication of the Low strain is possible in the closing part of the path even when no one in the Low strain is treated. Since this is feasible, it is also optimal, as it is cheaper than treating unnecessarily.

### Optimal policy when $K<1$

In this section we address the case when $K<1$. In this regime, it is possible to have equilibria where both strains of the disease prevail. The parameter values are given in Table 5.

**Table 5:** Parameter values for K>1

Parameter	Value
$\beta _{H}$	0.8
$\beta _{L}$	0.4
$\tau$	0.15
$\alpha$	0.2
*p*	1
$\delta$	0.222

**Fig. 7:**
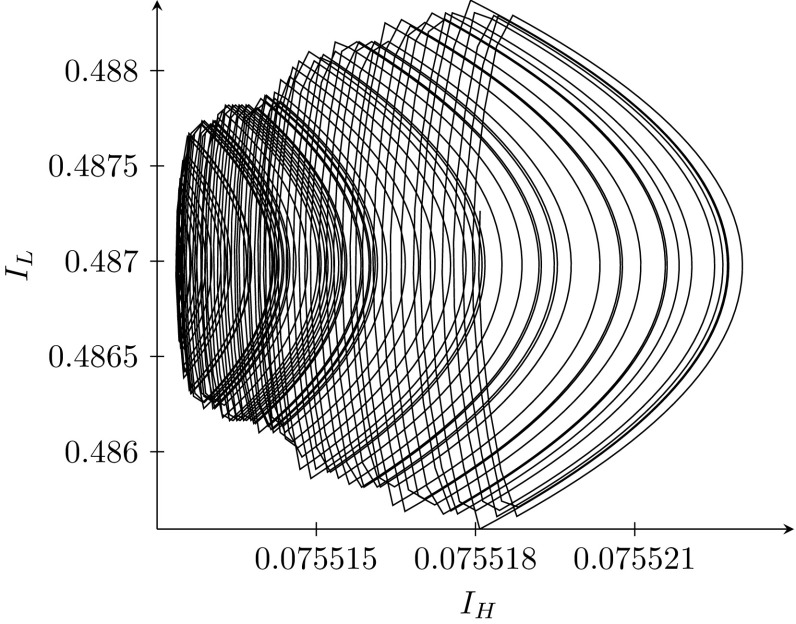
Evolution of prevalence along the path towards equilibrium $F^{low}$, with $I_{H}^{0}=0.0755,$ $I_{L}^{0}=$ 0.4870 and $c=0.4$. This path is simulated only backwards

We simulate optimal policy in the region of one of the non-boundary fixed points. In particular, we assume that it is optimal to go to the fixed point $F^{low}$, and simulate optimal policy backwards, starting from that fixed point. The evolution of prevalence is shown in Fig. 7.

**Fig. 8:**
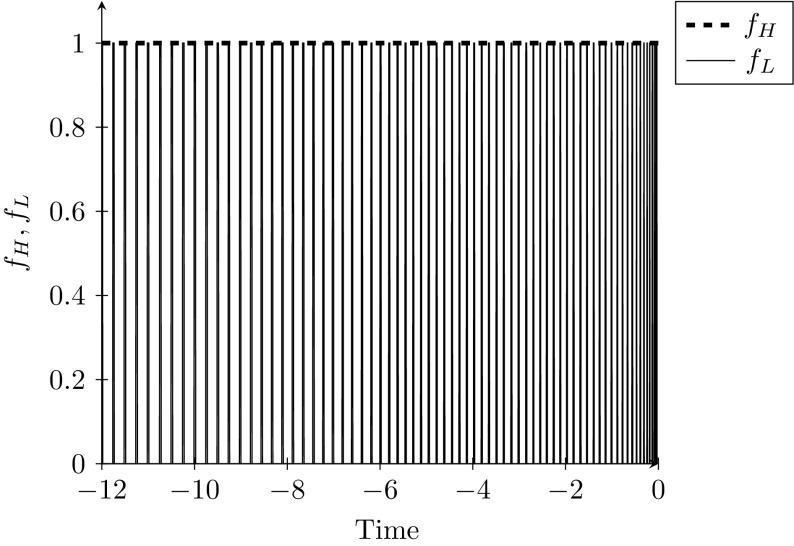
Evolution of optimal policy along the path towards equilibrium $F^{low}$, with $I_{H}^{0}=0.0755,$ $I_{L}^{0}=$ 0.4870 and $c=0.4$. This path is simulated only backwards

This figure clearly shows a spiral. These simulations suggest that non-boundary fixed points are never the end points of optimal paths. In every case examined, perturbing the fixed point solution and running the path backwards generates a high frequency cycle. Reversing the path from its end point generates an integral which is exceedingly large. Figure 8 shows the evolution of optimal policy along this same path. The treatment of the Low strain switches between zero and one many times. Therefore, we conclude that even in the case when $K<1$, it is optimal to move to one of the boundary fixed points. It appears that coexistence of the two strains is never optimal.

### Chlamydia trachomatis

We began this paper with the example of chlamydia trachomatis, which is a disease with three strains that satisfies the behaviour of an SIS infection. As accurate modelling of the evolution of chlamydia trachomatis has been done in detail in the literature (e.g. Heijne et al. 2011; Sharomi and Gumel 2009; De Vries et al. 2006), we focus here on the distinction between the various strains, and the calculation of the cut-off parameter *K*, which is the novel contribution of this paper. In Table 6, we present the parameters that characterise chlamydia (serovars D-K) and LGV (serovars L1–L3) in the context of our SIS model with two strains.

**Table 6:** Parameters characterising chlamydia trachomatis

Parameter	Value	Source
$\beta _{H}$ (LGV)	0.60	N/A
$\beta _{L}$ (Chlamydia)	0.55	Althaus et al. (2012)
$\tau$	0.18	Geisler et al. (2008)
$\alpha$	0.97	Lau and Qureshi (2002)
*K*	0.02	
Eradicate High strain?	Yes	
Eradicate Low strain?	Yes	

There are challenges to estimating the right parameter values for diseases such as chlamydia and LGV. Due to a lack of studies on LGV, we use the recovery rate and treatment success rate of chlamydia. The transmission parameter depends on the number of contacts between the infected partner and the non-infected partner, and we use the per-partnership parameter estimated by Althaus et al. (2012). We were unable to find an estimate of the transmission parameter for LGV, but we know that it is higher than that for chlamydia, so we set it marginally higher at 0.6. Next, the spontaneous recovery parameter is challenging to measure because of the ethical considerations of not treating individuals diagnosed with chlamydia (Geisler 2010). Geisler et al. (2008) screened individuals for chlamydia and then screened them again when they came for a treatment visit, for which the median was 13 days later, where they found spontaneous resolution in 18% of patients. Finally, the treatment success probability faces the challenge of non-compliance (Bachmann et al. 1999). We use the measured success rate of treatment with azithromycin in a meta-analysis of randomised control trials, which is found to be 97% (Lau and Qureshi 2002).

These parameters imply two observations about chlamydia trachomatis in our model: first, the value of *K* is less than one, so that the full range of fixed points should be attainable—those that involve coexistence of chlamydia and LGV, and those that involve asymptotic eradication of chlamydia and positive prevalence of LGV, although our simulations suggest that coexistence is not the end of an optimal path. Second, and more importantly, both diseases should be possible to eradicate, if everyone who is infected is treated. The question then is why the prevalence of chlamydia, for example, is around 6% among people younger than 25 in the UK (Macleod et al. 2005). The likely reason is that chlamydia is often an asymptomatic disease: 75-85% women are asymptomatic, while over 50% of men are asymptomatic (Risser et al. 2005). Thus, the treatment parameter is far from equal to one. In fact, a rough back-of-the envelope calculation, assuming values of $I_{H}^{*}=0.001,I_{L}^{*}=0.06$ and the fact that total prevalence in equilibrium is given by Eq. (3) implies a treatment parameter of $f_{L}=0.35$.^16^ This suggests that only 35% of individuals infected with chlamydia at any given time are treated.

## Conclusion

We have derived analytically the fixed points in an SIS model with two strains, where neither superinfection nor eradication of the strains are possible, and the policymaker has access to two separate treatment instruments. We have characterised two types of fixed points: non-boundary fixed points, where the two strains co-exist, and boundary fixed points, which involve the asymptotic eradication of one strain. The feasibility of these fixed points depends on parameter values: we have defined the parameter *K*, which delineates three regimes of feasibility. For $K>1$, the only attainable equilibria are those where the Low strain is asymptotically eradicated and the High strain prevails. For $K\le 1$, the coexistence of the two strains is a feasible equilibrium.

The simulations present two interesting results. First, when $K>1$, optimal policy along the path to equilibrium exhibits switch points. This implies that a fixed policy is suboptimal. Further, in the neighbourhood of the equilibrium in our example, it is optimal to treat no one. This still allows the eradication of the Low strain. Second, when $K<1$, we simulate optimal policy in the neighbourhood of one of the non-boundary fixed points. We find that optimal policy exhibits a spiral. This suggests that this equilibrium can never be the end point of an optimal path, and hence that coexistence, although feasible, is unlikely to be optimal. This suggests that for all values of *K*, the welfare-maximising policy is one that leads to one of the asymptotic equilibria.

Further research should consider extending this model to include protection via vaccination as another instrument available to the policymaker.

## A Index of notation and acronyms

Table 7 defines the notation and acronyms used in this paper.

**Table 7:** Index of notation and acronyms

Notation	Definition
$j=H,L$	High, Low infectivity strain
$I_{j}(t)$	The prevalence of strain *j* in the population at time *t*
$I_{j}^{0}$	The initial prevalence of strain *j*
$f_{j}$	The proportion of individuals infected with strain *j* that are treated
$\beta _{j}$	The probability that a healthy person encountering a strain *j* infectee will become infected
$\alpha$	The success rate of treatment
$\tau$	The natural rate of recovery from the infection
*c*	The marginal cost of treatment
*p*	The value of a healthy individual in social welfare
$\delta$	The discount rate
$\lambda _{j}$	The multiplier in the Hamiltonian on the equation of motion for infection *j*
$F^{high}$	Fixed point where $f_{H}^{*}=\frac{(\beta _{H}-\beta _{L} )}{\beta _{L}}\frac{\tau }{\alpha }$ and $f_{L}^{*}=0$
$F^{low}$	Fixed point where $f_{H}^{*}=1$ and $f_{L}^{*} =1-\frac{(\beta _{H}-\beta _{L})}{\beta _{H}}\frac{\tau +\alpha }{\alpha }$
$F^{int}$	Continuum of fixed points where $f_{H}^{*}=1$ and $f_{L}^{*}=0$
$A_{L}^{high}$	The asymptotic fixed point where *H* is asymptotically eradicated, $f_{H}^{*}=1$ and $f_{L}^{*}=0$
$A_{H}^{high}$	The asymptotic fixed point where *L* is asymptotically eradicated, $f_{H}^{*}=0$ and $f_{L}^{*}=\in \{0,1\}$
$A_{H}^{low}$	The asymptotic fixed point where *L* is asymptotically eradicated, $f_{H}^{*}=1$ and $f_{L}^{*}=1$
*Z*	The set of feasible equilibria
*K*	Equal to $\frac{\beta _{H}-\beta _{L}}{\beta _{L}}\frac{\tau }{\alpha }$, the parameter that characterises regimes of feasibility
$P^{ab}$	The treatment policy $f_{H}=a,f_{L}=b$
AFP	Asymptotic fixed point
FP	Fixed point
MRAP	Most Rapid Approach Path

## B Asymptotic eradication

### Proposition 1

Both variants of the disease cannot be simultaneously eradicated even asymptotically in equilibrium if

$$\begin{aligned} \frac{\tau +\alpha }{\beta _{H}}<1,\\ \frac{\tau +\alpha }{\beta _{L}}&<1, \end{aligned}$$

which hold as a consequence of Assumption (4).

### Proof

To see this, first consider the case of the interior fixed points. Here, it is trivial. In the case of $F^{low}$, $I_{H}^{*}+I_{L}^{*}=1-\frac{\alpha +\tau }{\beta _{H}}$. It is not possible for $I_{H}^{*}+I_{L}^{*} =0$. Similarly for $F^{high}$ where $I_{H}^{*}+I_{L}^{*}=1-\frac{\tau }{\beta _{L}}$, it is not possible to have $I_{H}^{*}+I_{L}^{*}=0$. Next, consider the asymptotic fixed points. In the case of $A_{L}$, it is known that $I_{L}^{*}\rightarrow 0$ so the question is what happens to $I_{H}^{*}$. For $I_{L}$ to tend towards zero asymptotically, the necessary condition is $I_{H}>1-\frac{\alpha f_{H}+\tau }{\beta _{L}}$. Clearly $I_{H}\ne 0$ is necessary for this to be satisfied. Similarly, fixed point $A_{H}$ implies that $I_{H}^{*}\rightarrow 0$. The necessary condition for this is $I_{L}>1-\frac{\alpha f_{L}+\tau }{\beta _{H}}$, which can only be satisfied if $I_{L}\ne 0.$ Thus, both variants of the disease cannot be eradicated in equilibrium, even asymptotically. $\square$

## C Feasible policy along the path to equilibrium

In order to derive which policies are feasible along the path towards equilibrium, it is necessary to examine each MRAP policy in turn and derive the conditions that are required for $I_{H}$ and $I_{L}$ to either increase or decrease.

First, consider $P^{00}$. This policy implies

$$\begin{aligned} \frac{\dot{I}_{H}}{I_{H}}&=\beta _{H}(1-I_{H}-I_{L})-\tau ,\\ \frac{\dot{I}_{L}}{I_{L}}&=\beta _{L}(1-I_{H}-I_{L})-\tau . \end{aligned}$$

There are two ways of approaching a fixed point with this policy. First, it is possible that $\dot{I}_{H},\dot{I}_{L}>0$ (i.e. $I_{H}$ and $I_{L}$ are increasing towards $I_{H}^{*}$ and $I_{L}^{*}$). The required conditions for this are

$$\begin{aligned} 1-\frac{\tau }{\beta _{L}}>I_{H}+I_{L},\\ 1-\frac{\tau }{\beta _{H}}&>I_{H}+I_{L}, \end{aligned}$$

which collapse to

$$\begin{aligned} 1-\frac{\tau }{\beta _{L}}>I_{H}+I_{L}. \end{aligned}$$

Similarly, it is possible that $\dot{I}_{H},\dot{I}_{L}<0$ (i.e. $I_{H}$ and $I_{L}$ are decreasing towards $I_{H}^{*}$ and $I_{L}^{*}$). The required conditions for this are

$$\begin{aligned} 1-\frac{\tau }{\beta _{L}}<I_{H}+I_{L},\\ 1-\frac{\tau }{\beta _{H}}&<I_{H}+I_{L}, \end{aligned}$$

which collapse to

$$\begin{aligned} 1-\frac{\tau }{\beta _{H}}<I_{H}+I_{L}. \end{aligned}$$

Next, consider $P^{10}$. For $\dot{I}_{H},\dot{I}_{L}>0$, the required conditions are

$$\begin{aligned} 1-\frac{\tau }{\beta _{L}}>I_{H}+I_{L},\\ 1-\frac{\tau +\alpha }{\beta _{H}}&>I_{H}+I_{L}, \end{aligned}$$

where the overriding condition is

$$\begin{aligned} 1-\frac{\tau }{\beta _{L}}>I_{H}+I_{L}. \end{aligned}$$

For $\dot{I}_{H},\dot{I}_{L}<0$, the required conditions are,

$$\begin{aligned} 1-\frac{\tau }{\beta _{L}}<I_{H}+I_{L},\\ 1-\frac{\tau +\alpha }{\beta _{H}}&<I_{H}+I_{L}, \end{aligned}$$

both of which are satisfied when

$$\begin{aligned} 1-\frac{\tau +\alpha }{\beta _{H}}<I_{H}+I_{L}. \end{aligned}$$

Third, take $P^{11}$. For $\dot{I}_{H},\dot{I}_{L}>0$, it needs to be the case that

$$\begin{aligned} 1-\frac{\tau +\alpha }{\beta _{H}}>I_{H}+I_{L},\\ 1-\frac{\tau +\alpha }{\beta _{L}}&>I_{H}+I_{L}, \end{aligned}$$

where the overriding condition is

$$\begin{aligned} 1-\frac{\tau +\alpha }{\beta _{L}}>I_{H}+I_{L}. \end{aligned}$$

For $\dot{I}_{H},\dot{I}_{L}<0$, it is required that

$$\begin{aligned} 1-\frac{\tau +\alpha }{\beta _{H}}<I_{H}+I_{L},\\ 1-\frac{\tau +\alpha }{\beta _{L}}&<I_{H}+I_{L}, \end{aligned}$$

both of which are satisfied when

$$\begin{aligned} 1-\frac{\tau +\alpha }{\beta _{H}}<I_{H}+I_{L}. \end{aligned}$$

Last, consider $P^{01}$. For $\dot{I}_{H},\dot{I}_{L}>0$, it needs to be the case that

$$\begin{aligned} 1-\frac{\tau }{\beta _{H}}>I_{H}+I_{L},\\ 1-\frac{\tau +\alpha }{\beta _{L}}&>I_{H}+I_{L}. \end{aligned}$$

where the overriding condition is

$$\begin{aligned} 1-\frac{\tau +\alpha }{\beta _{L}}>I_{H}+I_{L}. \end{aligned}$$

For $\dot{I}_{H},\dot{I}_{L}<0$, it is required that

$$\begin{aligned} 1-\frac{\tau }{\beta _{H}}<I_{H}+I_{L},\\ 1-\frac{\tau +\alpha }{\beta _{L}}&<I_{H}+I_{L}, \end{aligned}$$

both of which are satisfied when

$$\begin{aligned} 1-\frac{\tau }{\beta _{H}}<I_{H}+I_{L}. \end{aligned}$$

## Footnotes

1 Centers for Disease Control and Prevention Morbidity and Mortality Weekly Report (April 1 2011) and Public Health England, Infection Report, Volume 9 Number 22.

2 Lyme Disease Association of Australia (http://lymedisease.org.au).

3 Centers for Disease Control and Prevention, http://www.cdc.gov.

4 More information on this can be found at the Centers for Disease Control and Prevention (http://www.cdc.gov/hiv/topics/basic) as well as the charity AVERT (http://www.avert.org/hiv-types.htm).

5 See Keeling and Eames (2005) and Bansal et al. (2007) for a discussion of how mixing networks can be applied to the study of epidemiology.

6 For more detail on these concepts, the reader is referred to Skiba (1978) and Spence and Starrett (1975).

7 An index of notation and acronyms used in the paper can be found in Appendix A.

8 Other assumptions are possible; for example, a policymaker may know an individual is ill but not which strain of the disease he has. In this case, the policymaker would effectively have only one policy instrument at hand. Manski (2013) discusses treatment under uncertainty.

9 Note that $\lambda _{H}$ and $\lambda _{L}$ are always negative, as an additional infected person in the population lowers social welfare. I write the condition here in absolute values to ease interpretation.

10 This result depends on the assumption that the success rate of treatment $\alpha$ and the natural rate of recovery $\tau$ are the same between the two strains. If $\alpha$ or $\tau$ were substantially higher for the High strain than the Low strain, this result could be reversed, with more treatment mandated for the Low strain. See Sect. 4.5.

11 These figures are illustrated for the following parameter values: $\beta _{L}=0.4,$ $\tau =0.15$ and $\alpha =0.2$, with $\beta _{H}=0.95$ for $K>1$, $\beta _{H}=0.9\dot{3}$ for $K=1$, and $$\beta _{H}=0.8\,\ $$for $K<1$.

12 In our simulations, we also explored the properties of “singular solutions”. A singular solution is a path along which one of the control variables takes an interior value (i.e. strictly less than one and greater than zero). Such paths were not optimal in the cases we examined.

13 Note that when $f_{H}^{*}=1$, $I_{H}^{*}=1-\frac{\tau +\alpha }{\beta _{H}}=0.6316$. When $f_{H}^{*}=0$, $I_{H} ^{*}=1-\frac{\tau }{\beta _{H}}=0.8421$.

14 All of these simulations show the same qualitative results when the initial prevalence of the Low strain is set to a smaller value: $I_{L}^{0}=0.01,I_{L}^{0}=0.001$ and $I_{L}^{0}=0.0001$.

15 The backward paths are necessarily Hamiltonian paths as long as the Hamiltonian conditions for the forward paths are satisfied. This is because the backward paths are merely a continuation, albeit in the opposite direction, of the Hamiltonian paths.

16 This is implied by an equilibrium prevalence of $I_{H}+I_{L}=1-\frac{\tau +\alpha f_{L}}{\beta _{L}}$. The prevalence of LGV is very low: Ward et al. (2007) estimate that there were only 327 cases in the UK between 2004 and 2006. We assume a prevalence of 0.001 but setting this number closer to zero would not have a substantive impact on our estimate of $f_{L}$.
